# Sequential targeting of PARP with carboplatin inhibits primary tumour growth and distant metastasis in triple-negative breast cancer

**DOI:** 10.1038/s41416-023-02226-w

**Published:** 2023-03-20

**Authors:** Michèle Beniey, Audrey Hubert, Takrima Haque, Alexia Karen Cotte, Nelly Béchir, Xiaomeng Zhang, Danh Tran-Thanh, Saima Hassan

**Affiliations:** 1 Department of Surgery, Université de Montréal, Montreal, QC Canada; 2 Institut du cancer de Montréal, Centre de Recherche de Centre hospitalier de l’Université de Montréal (CRCHUM), Montréal, QC Canada; 3 Université de Montréal, Montréal, QC Canada; 4 Department of Pathology, Centre hospitalier de l’Université de Montréal (CHUM), Montreal, QC Canada; 5 Division of Surgical Oncology, CHUM, Montreal, QC Canada

**Keywords:** Breast cancer, Targeted therapies

## Abstract

**Background:**

Patients with triple-negative breast cancer (TNBC) develop early recurrence. While PARP inhibitors (PARPi) have demonstrated potential in *BRCA1/2*-mutant (BRCA^MUT^) TNBC, durable responses will likely be achieved if PARPi are used in combination. It is plausible that sequential administration of a potent PARPi like talazoparib in combination with carboplatin can enhance primary tumour and metastasis inhibition in BRCA^MUT^ and BRCA1/2 wild-type (BRCA^WT^) TNBCs, and decrease toxicity.

**Methods:**

We evaluated the impact of the concurrent combination of talazoparib and carboplatin on cell survival in 13 TNBC cell lines. We compared the concurrent and sequential combination upon fork replication, migration and invasion. We also used three orthotopic xenograft models to evaluate primary tumour growth, distant metastasis, and toxicity.

**Results:**

Concurrent talazoparib and carboplatin was synergistic in 92.3% of TNBC cell lines, independent of *BRCA1/2*-mutation status. The sequential combination decreased fork speed in normal cells, but not in TNBC cells. The talazoparib-first sequential combination resulted in a strong reduction in migration (70.4%, *P* < 0.0001), invasion (56.9%, *P* < 0.0001), lung micrometastasis (56.4%, *P* < 0.0001), and less toxicity in a BRCA^WT^ model.

**Conclusion:**

The sequential combination of talazoparib and carboplatin is an effective approach to inhibit micrometastatic disease, providing rationale for the use of this combination in early TNBC patients.

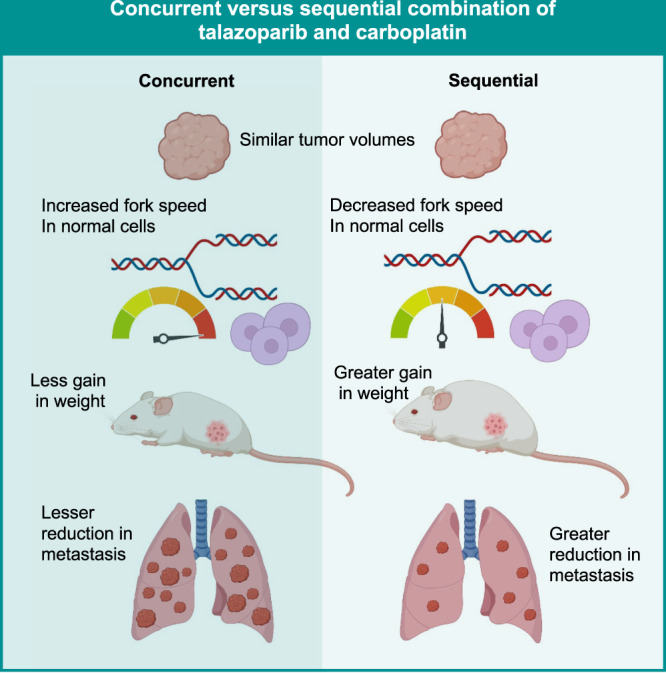

## Background

Triple-negative breast cancer (TNBC) is the most aggressive subtype of breast cancer with the poorest outcomes. Representing 15–20% of all breast cancers, TNBCs lack oestrogen/progesterone receptor (ER/PR) and human epidermal growth factor receptor 2 (HER2) overexpression. Most patients receive four or five types of chemotherapy yet still suffer from rapid disease progression with significant toxicity. An orally available family of targeted therapeutic agents, Poly (ADP-Ribose) Polymerase inhibitors (PARPi), was approved by the FDA for metastatic and early TNBC patients with germline mutations in *BRCA1/2* (gBRCA^MUT^). However, patients with gBRCA^MUT^ constitute about 11–20% of all TNBCs [1–4].

The efficacy of PARPi monotherapy in gBRCA^MUT^ patients was demonstrated in the metastatic, neoadjuvant (pre-surgery), and adjuvant (post-surgery) settings [5–8]. In particular, talazoparib and olaparib demonstrated an improvement in progression-free survival (PFS) and less toxicity in comparison to standard chemotherapy in the metastatic setting [5, 6]. Talazoparib was also associated with a pathologic complete response (pCR) in 53% of gBRCA^MUT^ patients in the neoadjuvant context [7]. Importantly, in the adjuvant setting, in comparison to placebo, olaparib improved 3-year distant disease-free survival (87.5% versus 80.4%) [8], suggesting that olaparib can inhibit the development of micrometastatic disease.

PARPi target PARP1/2 enzymes and have two main mechanisms of action: synthetic lethality and PARP-DNA trapping [9, 10]. Synthetic lethality occurs in the context of *BRCA1/2* mutations, whereby PARPi prevent the release of PARP1 from DNA, leading to an accumulation of double-stranded DNA breaks, resulting in complex chromatid rearrangements and cell death [11, 12]. Trapped PARP-DNA complexes form DNA lesions that are not bypassed by replication forks and induce cytotoxicity [10, 13, 14]. While various PARPi demonstrate similar catalytic activity, they differ in their capacity for PARP-DNA trapping, with the most potent PARPi being talazoparib, then niraparib, followed by olaparib, and finally veliparib [15].

Despite PARP1 being widely known for its activities in DNA repair, PARP1 has also been implicated in several other cancer cell functions [16]. PARP1 regulates chemokine signalling, facilitating tumour dissemination at several key steps of metastasis, including angiogenesis, adherence of tumour cells to endothelium, cell migration, and cancer cell extravasation at the metastatic site [17]. PARP1 has also been shown to promote lung metastasis using a mechanism independent of DNA repair [18]. Furthermore, alterations in DNA repair genes in distant metastasis have also been detected in melanoma and colon cancer [19], all pointing towards a plausible role of PARPi in inhibiting the development of distant metastasis through DNA repair-dependent and independent mechanisms.

While PARPi have mainly been used in gBRCA^MUT^ breast cancer patients, recent clinical trials have observed the efficacy of PARPi beyond patients with gBRCA^MUT^ [2, 20, 21]. We and others have demonstrated preclinical efficacy of PARPi in TNBCs that are both BRCA^MUT^ and *BRCA1/2* wild type (BRCA^WT^) [22–24]. Efficacy in BRCA^WT^ tumours is likely due to BRCAness, a phenotype similar to BRCA^MUT^, with a defect in homologous recombination repair, but actually lack the mutation in *BRCA1/2* [25]. However, one of the clinical challenges is that patients treated with PARPi monotherapy often develop therapeutic resistance.

Hence, to improve overall survival, it is likely that PARPi will need to be administered in combination. Carboplatin, an alkylating agent, is one such promising chemotherapeutic agent. While one trial did not demonstrate the added benefit of low-dose veliparib to carboplatin and chemotherapy [26], higher-dose veliparib in combination with carboplatin and paclitaxel resulted in a more durable response in BRCA^MUT^ metastatic patients [27]. Little is known about the role of the combination of a potent PARPi plus carboplatin in preventing the development of metastatic breast cancer in BRCA^WT^ patients.

While toxicity was a concern with the concomitant and continuous administration of talazoparib and carboplatin [28], it is plausible that a sequenced and intermittent dosing regimen may decrease toxicity. Pre-treatment with carboplatin was previously shown to be a plausible approach to increase cellular uptake of olaparib and increase olaparib clearance, which may improve efficacy and decrease toxicity [29]. Furthermore, lower endogenous replication stress in normal cells, in comparison to cancer cells, helped to explain the increased toxicity observed with concomitant PARPi combination with a targeted therapeutic agent [30].

In this study, our goals were to determine the impact of different dosing strategies of the combination of a potent PARPi, talazoparib and carboplatin, on tumour efficacy, toxicity, and development of metastasis. We accomplished this by using a panel of TNBC cell lines, characterising cell proliferation, the sustained DNA damage response and apoptosis. We further evaluated the replication fork speed and DNA damage response in both TNBC and normal cells. Moreover, we utilised three orthotopic xenograft models, including BRCA^MUT^ and BRCA^WT^ tumours, to evaluate the influence of different sequencing strategies on primary tumour growth, mice weight, haematologic toxicity, and distant metastasis. We additionally evaluated the impact of the sequencing strategies on cell migration, invasion, differential expression of chemokines, and gene expression in metastatic lung tissue.

## Materials and methods

A detailed list of all reagents and resources, including cell lines with sources, are provided in Supplementary Methods Table S1.

### Cell lines

All cell lines were validated by DNA fingerprinting using short-term repeat (STR) analysis done by Genome Quebec (microsatellite geneprint 10) (Montreal, Canada), last performed March 2021, and were mycoplasma free, tested using Mycoalert mycoplasma detection kit (LT07, Lonza).

Ten-day chemosensitivity assay and immunofluorescence staining, analysis and visualisation were performed as previously described [22] and explained in Supplementary Methods.

### High-content imaging

High-content imaging was done using Operetta (PerkinElmer) with a ×20 objective and filter sets for Alexa 488, Alexa 647, and DAPI. We scanned 47 images per well and performed image analysis with Harmony High-Content Imaging and Analysis Software (version 4.1, PerkinElmer Inc.).

### Combination index values

Cells were treated with six concentrations with twofold dilutions of either carboplatin, talazoparib alone or concomitant combination using the 10-day chemosensitivity assay. Cells were fixed, stained with DAPI, imaged using Operetta (PerkinElmer), and enumerated using Harmony High-Content Imaging and Analysis Software (version 4.1, PerkinElmer Inc.). Combination Index (CI) and Dose Reduction Index (DRI) values were calculated at Fa = 0.50, using the Chou-Talalay method with Compusyn software (ComboSyn Incorporated, Paramus, NJ, USA). CI values between 0.9 and 1.10 are considered nearly additive; 0.85–0.7 demonstrate moderate synergism, and 0.3–0.7 indicate synergism [31].

See Supplementary Methods for details regarding drug treatment strategies.

### Flow cytometry

Cells were fixed/permeabilized in ice-cold 70% ethanol overnight. Samples were washed, blocked with PBS, Triton 0.2%, BSA 1% solution for 15 min at room temperature. Cells were then incubated with gamma H2AX-AlexaFluor 647 (1:100; #613408, BioLegend), phospho-Histone H3- AlexaFluor 488 (Ser-10) (1:100; #3465, Cell Signaling), cleaved-caspase 3-Pacific Blue™ (1:100; #8788, Cell Signaling), and 20 µg/mL propidium iodide (PI) (#P3566, Invitrogen) with 200 µg/mL RNase A (#10109142001, Roche®), for 1 h at room temperature. Samples were analysed with FACS BD LSRFortessa™ cell analyzer (BD Biosciences). At least 10,000 events were assessed per measurement. Staining was analysed with FlowJo™ (BD Biosciences). Values were obtained from triplicate assays performed in triplicate wells. Gating strategy is presented in Supplementary Methods.

### DNA fibre assay

Cells were sequentially labelled with 25 µM of 5-Chloro-2′-deoxyuridine (CldU) (#C6891, Sigma-Aldrich) and then 250 µM of 5-Iodo-2’-deoxyuridine (IdU) (#I7125, Sigma-Aldrich) for 30 min each [32]. Cells were resuspended in ice-cold PBS at 5 × 10^5^ cells/mL. Four drops of 2.5 µL of cell suspension was pipetted in staggered rows onto a microscope slide (Cat #4951PLUS602811, Fisherbrand™). In all, 6 µL of spreading buffer (200 mM Tris-HCl, 50 mM EDTA, 0.5% SDS, pH 7.4) was added to cell suspension drops. DNA was allowed to run down the slide, slowly tilting slides at 15–45°. DNA was air-dried and fixed in methanol/acetic acid (3:1) for 10 min. DNA was denatured in 2.5 M HCl for 45 min. Slides were incubated in blocking solution (PBS, 2% BSA, 0.1% Tween) for 1 h, followed by rat anti-BrdU [clone BU1/75 (ICR1)] (1:100, #ab6326, Abcam) and Mouse anti-BrdU [clone B44] antibodies (1:20, #347580, BD Biosciences), overnight at 4 °C. Slides were incubated with anti-Rat AlexaFluor 568 (1:500, #A11077, Invitrogen) and anti-Mouse AlexaFluor 488 (1:500, #A21202, Invitrogen) antibodies, for 1 h at room temperature. Slides were mounted in ProLong™ Gold antifade Mountant (#P36930, Invitrogen) and analysed using a Zeiss Axio Observer Z1 microscope with ×63 oil objective. Replication fork speed was calculated using the CldU + IdU track length/60 min * 2.59 kb/µm.

### Orthotopic xenografts

All animal experiments were approved by the Institutional Animal Protection Committee (CIPA) of the Centre de Recherche de Centre hospitalier de l’Université de Montréal (CRCHUM) under protocol C17017SHs. Either 5 million MDAMB231 or 2 million MX1/HCC1806 cells were resuspended in 50% Matrigel Matrix Phenol Red Free (#CB40234C, Fisher Scientific), 25% PBS and 25% collagen type 1 (#08-115, Millipore Sigma) solution [33]. Using 7-week-old NOD-SCID gamma (NSG) female mice (#005557, Jackson Laboratory), 0.2 mL cell suspension was surgically implanted. Once tumours reached an average volume of 150 mm^3^, mice were randomised into treatment groups based on tumour volume volumes and weight. Our sample size was based on the experiments previously described [34]. Except for the control group for MDAMB231, which had 14 mice, each treatment group consisted of 8–10 mice. See Supplementary Methods for further details.

### Cell migration and invasion assays

In all, 3 × 10^4^ or 5 × 10^4^ cells were suspended in serum-free media in 8-μm transwell inserts (#83.3932.800, Sarstedt), for 24-h migration or 48-h invasion assays, respectively. For the invasion assay, the upper chamber was pre-coated with 1/9 dilution of Matrigel (Cultrex Reduced Growth Factor Basement Membrane Extract, #3433-005-01, R&D Systems). After incubation, inserts were washed with PBS. Migrated or invaded cells on the underside of the membrane were fixed with 4% paraformaldehyde and stained with hematoxylin (Hematoxylin QS Counterstain, #H-3404, Vector Laboratories). Migrated or invaded cells were counted from six representative fields from the inverted EVOS XL core microscope (Thermo Fisher Scientific).

### Chemokine array and ELISAs

In total, 200 μg of protein was extracted from cell lysates from MDAMB231 cells to detect the expression of 31 chemokines using the human chemokine array kit (#ARY017, R&D Systems). Images of membranes were acquired with ChemiDoc (BioRad) and dots were quantified using QuickSpots (Western vision) software. At the same time point, supernatants of MDAMB231 and HCC1395 cells were collected to detect MCP-1 levels with the human CCL2/MCP-1 Immunoassay (#SCP00, R&D Systems).

See Supplementary Methods for details regarding RNA-seq analysis.

### Statistical analysis

Normality of each dataset was first verified before performing Kruskal–Wallis ANOVA or one-way ordinal ANOVA with multiple-comparison post test. Two-way ANOVAs with post-tests were performed for grouped conditions. Data are represented as mean + /− SEM, except for fork speed, in which median values with interquartile ranges are presented. Data were analysed and plotted using GraphPad Prism software 8. *P* < 0.05 is considered statistically significant.

### Reporting summary

Further information on research design is available in the Nature Research Reporting Summary linked to this article.

## Results

### Talazoparib and carboplatin synergise in most TNBC cell lines

**Fig. 1:**
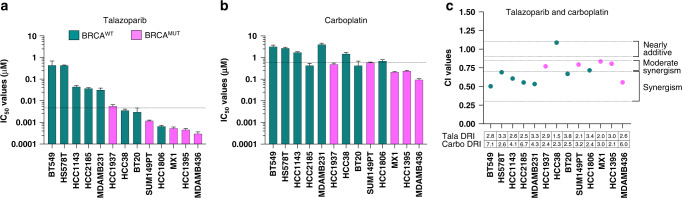
Talazoparib synergises with carboplatin in most TNBC cell lines. IC_50_ values for (**a**) talazoparib, and (**b**) carboplatin in TNBC cell lines. Data are mean + /− SEM. The dashed line indicates the median IC_50_ value for all cell lines for that therapeutic agent. Effect of the combination of talazoparib and carboplatin demonstrated in (**c**) by combination index values reported at an FA of 0.5. Under each cell line is the dose-reduction index (DRI) for talazoparib and carboplatin. Teal green bars/dots represent BRCA^WT^ cell lines, and pink bars/dots represent BRCA^MUT^ cell lines.

We determined the IC_50_ values of talazoparib and carboplatin as single agents in a panel of 13 TNBC cell lines (Fig. 1, Supplementary Table 1 and Supplementary Fig. 1). Talazoparib demonstrated a larger dynamic range, with IC_50_ values ranging from 0.0003 to 0.44 μM, in comparison to carboplatin, with IC_50_ values ranging from 0.09 to 4.0 μM (Fig. 1a, b). Using the median IC_50_ values, we set a threshold of 0.0036 μM for talazoparib and 1.25 μM for carboplatin to define sensitivity and resistance. Concurrent administration of talazoparib and carboplatin resulted in synergy, with Combination Index (CI) values <1 in 92.3% (12/13) of cell lines (Fig. 1c). Stronger synergy was observed mainly in BRCA^WT^ cell lines and those that were PARPi-resistant. Dose-reduction indices (DRI) were calculated to determine the dose-fold reduction of each therapeutic agent to achieve synergy. DRI for talazoparib ranged from 1.5 to 3.8, while the DRI for carboplatin ranged from 2.1 to 7.1. This is suggestive that synergy can be accomplished with important dose reductions of both drugs, with greater reductions in carboplatin versus talazoparib commonly observed in 61.5% (8/13) of cell lines.

### Lower concentrations of talazoparib and carboplatin required for DNA damage and cell death in PARPi-resistant TNBC cell lines

**Fig. 2:**
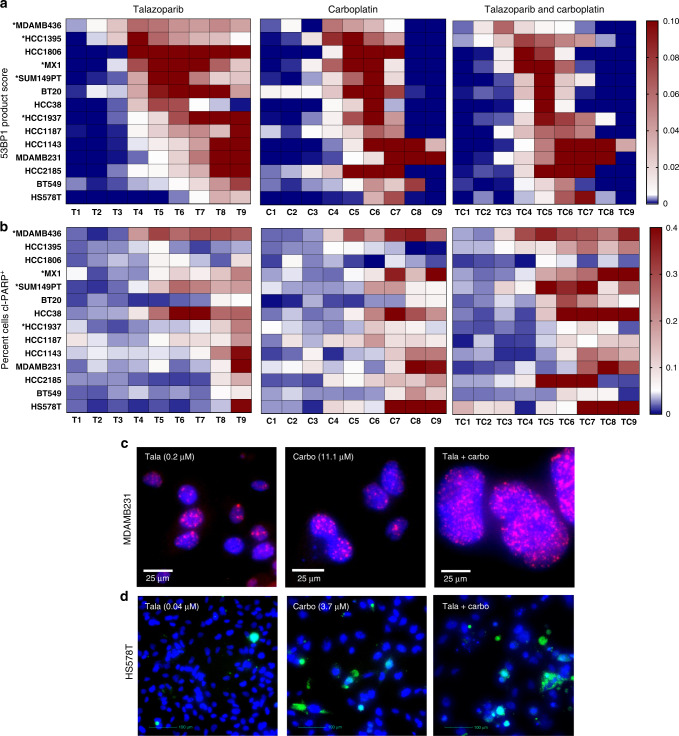
DNA damage and apoptosis induced at lower concentrations with the combination of talazoparib and carboplatin. Heatmaps of normalised values of either (**a**) 53BP1 product score (product of mean 53BP1 foci per nuclei and percentage of cells positive for 53BP1) (upper panel) and **b** percentage of cleaved (cl)-PARP positive (^+^) cells (second panel). Along *x* axis are increasing drug concentrations, 1–9, for talazoparib, T; carboplatin, C, and the concurrent combination of talazoparib and carboplatin, TC. Along the *y* axis are TNBC cell lines, of which * indicates BRCA^MUT^ cell lines. Representative images at 20x objective of 53BP1 foci in MDMAB231 are shown in (**c**), and cl-PARP^+^ cells in HS578T are shown in (**d**). MDAMB231 and HS578T cells are treated with talazoparib (tala) (left column), carboplatin (carbo) (middle column), and the combination of tala + carbo (right column). Blue represents nuclear staining, pink represents 53BP1 foci, and green represents cl-PARP.

We determined the impact of 9 increasing concentrations (1 refers to the lowest, and 9 refers to the highest concentration) of talazoparib (T1-9), carboplatin (C1-9), or the concomitant combination of talazoparib and carboplatin (TC1-9) upon 53BP1 response and apoptosis using a 10-day chemosensitivity assay in 14 TNBC cell lines (Fig. 2). DNA damage was quantified using a 53BP1 product score (Fig. 2a), calculated from the product of mean number of nuclear 53BP1 foci (Fig. 2c and Supplementary Fig. 2A) and percentage of cells positive for 53BP1. We identified a dose-dependent response of the 53BP1 product score to talazoparib. The combination of talazoparib and carboplatin had the greatest impact in the PARPi-resistant cell lines, where lower concentrations of each of the drug induced a DNA damage response that otherwise required higher drug concentrations as monotherapy. We also evaluated apoptosis by calculating percent cells positive for cleaved-PARP (cl-PARP^+^ ) (Fig. 2b, d and Supplementary Fig. 2B). While cell death required higher concentrations for talazoparib (mainly T8, 9) or carboplatin alone (mainly C7, 8, 9), combination therapy induced apoptosis at lower concentrations, starting at TC5, and was strongly present in 10/14 cell lines.

### Sequential combination comparable to concurrent combination in TNBC cells but decreases replication fork speed in normal cells

**Fig. 3:**
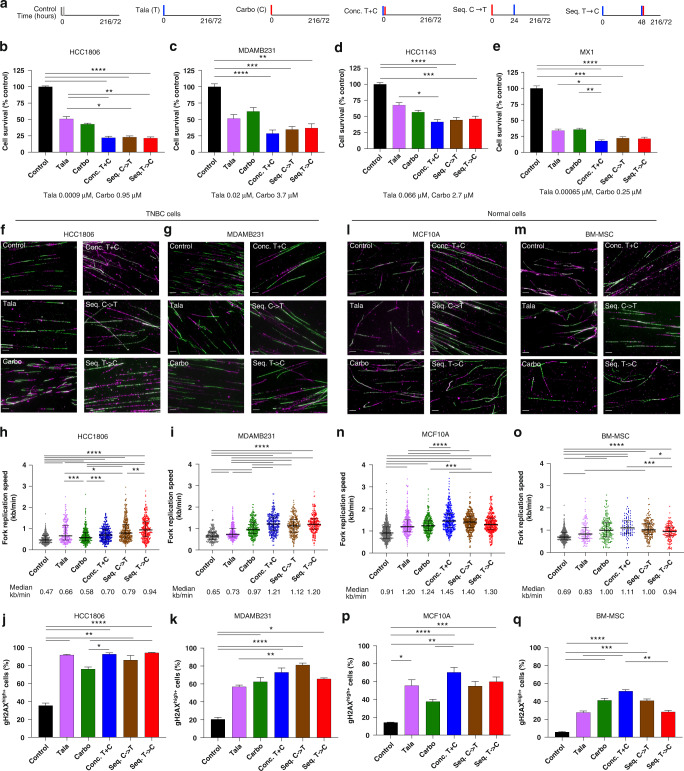
Differential effect of the sequential combination in TNBC and normal cells. Impact of the combination of talazoparib and carboplatin using concurrent or sequential approaches on cell proliferation, fork speed, and DNA damage. Using a 9-day (216-hour) treatment assay (**a**), cell survival was determined for **b** HCC1806, **c** MDAMB231, **d** HCC1143 and **e** MX1 TNBC cell lines. Using a 72-h treatment assay (**a**), impact of treatment on fork speed (DNA fibre assay) and DNA damage (flow cytometry) was shown in TNBC cell lines (**f**–**i**), and normal cell lines (**l**–**o**). **f**, **g**, **l**, **m** Representative images of DNA fibres with DNA labelling with CldU (magenta) and IdU (green) thymidine analogues. Images were taken at ×63 objective. Scale bars, 10 microns. **h**, **i**, **n**, **o** Dot plots of fork speed (kb/min), with a mean count of 313 DNA fibres/condition. Line represents median values with interquartile range. Impact of treatments on DNA damage represented by γ-H2AX^+^ cells in (**j**, **k**, **p**, **q**) Data are represented as mean +/– SEM. ANOVA with post test for multiple comparisons was performed. ****P < 0.0001; ***P < 0.001; **P < 0.01; *P < 0.05.

We first evaluated the impact of three different combination strategies including concurrent administration of talazoparib and carboplatin (conc.T + C) and two sequential approaches: carboplatin first followed by talazoparib (seq.C→T), and talazoparib run-in approach followed by carboplatin (seq.T→C) upon cell survival using the 10-day chemosensitivity assay (Fig. 3a). We selected three BRCA^WT^ cells, including two PARPi-resistant cell lines, HCC1143 and MDAMB231, a PARPi-sensitive yet carboplatin-resistant cell line, HCC1806; and one PARPi-sensitive cell line that is both *BRCA1*-deleted and *BRCA2-*mutated, MX1. In all four cell lines, cell survival was comparable between the concomitant and sequential combination approaches, with either talazoparib or olaparib as the PARPi backbone (Fig. 3b–e and Supplementary Fig. 7A, B).

To better understand the impact of the combination approaches on in vitro toxicity, we compared fork replication speed and DNA damage response between TNBC and normal cells. We used two TNBC cell lines, HCC1806 and MDAMB231, and two human normal cell lines including MCF10A (breast epithelial cell line) and BM-MSC (bone marrow-derived mesenchymal stem cells) (Fig. 3f–q). We determined that the mean endogenous replication fork speed was 0.56 kb/min for the TNBC cells in comparison to 0.8 kb/min for the normal cells.

Treatment with talazoparib for 72 h increased fork speed in all four cell lines, which is similar to what was previously reported when talazoparib was administered for 2 or 24 h [35, 36]. In comparison to control, the concomitant combination increased fork speed by 86.0% (*P* < 0.0001), 48.9% (*P* < 0.0001), 59.2% (*P* < 0.0001), and 59.9% (*P* < 0.0001) in MDAMB231, HCC1806, MCF10A and BM-MSC cells, respectively (Fig. 3h, i, n, o and Supplementary Figs. 3–6). The sequential approaches resulted in comparable or increased fork speeds in comparison to the concomitant approach in HCC1806 and MDAMB231. Similarly, there were no statistically significant differences in γ-H2AX^+^ cells between concurrent or sequential combination approaches in HCC1806, MDAMB231 (Fig. 3j, k), or MX1 (Supplementary Fig. 7D).

However, in normal cells, sequential treatments decreased fork speed in comparison to concomitant treatments. Seq.T→C demonstrated a 10.3% (*P* = 0.0004) and 14.8% (*P* = 0.0005) reduction in median fork speed in comparison to the concomitant combination, in MCF10A and BM-MSC cells, respectively. Conc.T + C resulted in the highest proportion of γ-H2AX^+^ cells in MCF10A (70.4%, *P* < 0.0001) and BM-MSC (51.4%, *P* < 0.0001) in comparison to control (Fig. 3p, q). In BM-MSC, seq.T→C reduced γ-H2AX + cells by 44.9% (*P* = 0.001), in comparison to conc.T + C. We also evaluated apoptosis in MCF10A and BM-MSC cells (Supplementary Fig. 7F, G). Of all the combination approaches, in comparison to control, conc. T + C resulted in the highest proportion of cl-Caspase 3 cells, with 43.8% (*P* < 0.0001), and 31.7% (*P* < 0.0001) for MCF10A and BM-MSC, respectively.

We and others previously demonstrated that PARPi resulted in S/G2 arrest [22, 30], and carboplatin also induced G2/M arrest [37, 38], and so we evaluated the impact of the combination on cell cycle changes. Greater accumulation of cells in G2 phases was observed with the concomitant and carboplatin-first combination approaches in MDAMB231, HCC1806, and MX1 (Supplementary Fig. 7H–L). Comparatively, treatment-induced cell cycle changes were less pronounced in normal cells in comparison to TNBC cells (Supplementary Fig. 7K, L), yet conc.T + C resulted in the greatest accumulation of cells in the S/G2 phases of BM-MSC, which was less distinct in the sequential approaches.

In summary, concurrent and sequential combination approaches resulted in similar cell proliferation, fork speeds and DNA damage responses in TNBC cells. However, in normal cells, sequential combination approaches resulted in decreased fork speed, decreased DNA damage and cell death.

### Concurrent and sequential combination have comparable primary tumour growth and tumour volume inhibition in three xenograft models

**Fig. 4:**
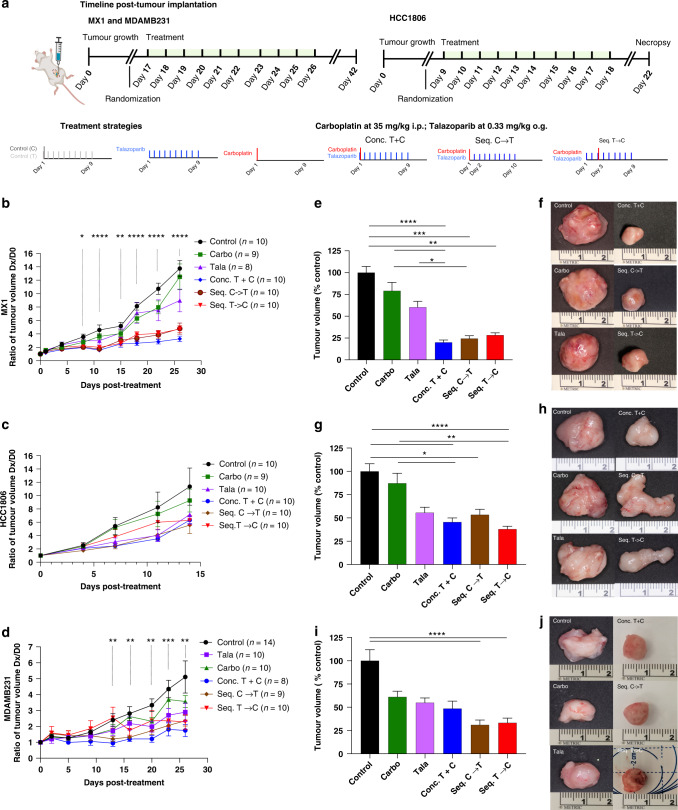
Comparable inhibition of primary tumour growth in vivo with concurrent and sequential combination strategies. Sequential versus concurrent combination treatment strategies were tested in three orthotopic xenografts with different tumour kinetics (**a**). Three TNBC models included MX1 (top row), HCC1806 (middle row), and MDAMB231 (bottom row). *N* = 8–14 mice per treatment group. Primary tumour growth rates are shown in (**b**, **c**, **d**). The *x* axis indicates days post treatment, where day 0 indicates the day before treatment. The *y* axis indicates normalised tumour volumes in which ratios of the tumour volumes on day x were divided by the tumour volumes on day 0 for that mouse. Ex vivo primary tumour volumes are quantified in (**e**, **g**, **i**). Data are represented as mean +/– SEM. ANOVA with post test for multiple comparisons was performed. ****P < 0.0001; ***P < 0.001; **P < 0.01; *P < 0.05. Representative images of ex vivo tumours are shown in (**f**, **h**, **j**). i.p. intraperitoneally, o.g. oral gavage.

To evaluate the impact of the combination approaches in vivo, we selected three orthotopic xenograft models comprising MX1, HCC1806 and MDAMB231 (Fig. 4a). In the MX1 and MDAMB231 xenografts, statistically significant differences in in vivo normalised tumour volumes were observed with different treatments from day 8 and day 13 until necropsy, respectively, while only trends were observed with HCC1806 (Fig. 4b–d).

At necropsy, combination approaches inhibited MX1 ex vivo primary tumour volumes by 79.9% (*P* < 0.0001), 75.5% (*P* = 0.0001), 71.7% (*P* = 0.002), by the conc.T + C., seq.C→T, and seq.T→C strategies, respectively, in comparison to control (Fig. 4e, f). For the HCC1806 xenograft, tumour volumes decreased by 54.4% (*P* = 0.002) with conc. T + C, 46.6% (*P* = 0.04) with seq.C→T, and 62.0% with seq.T→C (*P* < 0.0001) in comparison to control (Fig. 4g, h). For the MDAMB231 cohort, seq.C→T and seq.T→C demonstrated comparable effects with 68.9% (*P* < 0.0001), and 66.7% (*P* < 0.0001) reductions in tumour volume, respectively, which was more remarkable than the 51.4% reduction (*P* = 0.08) with conc.T + C, in comparison to control (Fig. 4i, j). There were no statistically significant differences in ex vivo primary tumour volumes between any of the combination groups within the three xenograft models.

### The sequential combination associated with less weight loss but no difference in haematologic toxicity in comparison to the concurrent combination in vivo

**Fig. 5:**
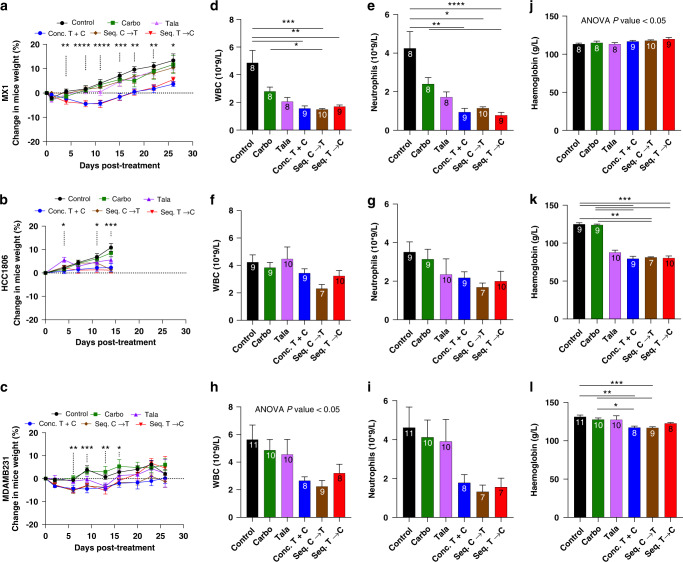
Sequential therapy can decrease weight loss in vivo with comparable haematologic toxicity as a concurrent combination. The impact of different treatment strategies was evaluated in three orthotopic xenograft models including MX1 (top row), HCC1806 (middle row) and MDAMB231 (bottom row). Kinetics of percent change in weight is shown in (**a**–**c**). Percent change in mice weight was calculated by normalising the weight on a given day by pre-treatment weight. *x* axis represents days post treatment until necropsy, and *y* axis represents percent change in weight. Impact of different treatments on white blood cell count (WBC) are shown in (**d**–**f**); neutrophil count shown in (**g**–**i**), and haemoglobin levels shown in (**j**–**l**). Sample sizes for each treatment are annotated at the top of each bar. Data are represented as mean +/– SEM. ANOVA with post test for multiple comparisons was performed. ****P < 0.0001; ***P < 0.001; **P < 0.01; *P < 0.05.

To evaluate toxicity, percent change in weight in comparison to pre-treatment weight was calculated for each orthotopic xenograft model (Fig. 5). The greatest decrease in weight by the combination approaches was demonstrated by MX1 and MDAMB231 cohorts, reaching a maximum of 5.2% decrease within 7 days of treatment. However, all mice gained weight by the time of necropsy, albeit 16 days (for MX1/MDAMB231) or 4 days (for HCC1806) after the termination of treatment. Nonetheless, at necropsy, the combination approach that consistently demonstrated minimal weight gain was conc.T + C, which was 3.7% for MX1, 2.3% for HCC1806, and 0.22% for MDAMB231 (Fig. 5a–c).

For the MX1 cohort, statistically significant changes in weight were first identified on day 4 post treatment, with smaller changes in weight gain demonstrated by conc.T + C and seq.T→C (Fig. 5a). At necropsy (16 days post treatment), in comparison to control, conc.T + C was the only group that differed in weight (*P* = 0.046), while seq.C→T demonstrated the greatest gain in weight at 10.4%. In the HCC1806 cohort (Fig. 5b), on the day of necropsy (4 days post treatment), weight gain was less than 3% for all combination approaches.

In the MDAMB231 cohort, one mouse that was treated with the conc.T + C approach had to be sacrificed 5 days prior to the rest of the cohort due to toxicity. The mouse was experiencing abdominal pain, with macroscopic and histologic analysis consistent with the diagnosis of haemorrhagic pancreatitis. On days 13 and 16 post treatment, seq. T→C demonstrated the greatest weight gain (4.6–6.7%) (Fig. 5c).

Haematologic toxicity was also evaluated in each xenograft model with plasma analysis at the time of necropsy. In the MX1 cohort, combination approaches decreased white blood cell counts with mean values of 1.6 (*P* = 0.002), 1.5 (*P* = 0.0003), and 1.7 (*P* = 0.0098), for conc.T + C, seq.C→T, and seq.T→C respectively, in comparison to control (mean 4.9) (Fig. 5d). Similarly, neutropenia was observed for conc.T + C (mean 0.95, *P* = 0.002), seq.C→T (mean 1.15, *P* = 0.01) and seq.T→C (mean 0.78, *P* < 0.0001), in comparison to control (mean 4.3) (Fig. 5e). No statistical significance was observed between the combination approaches. A similar trend, with greater leukopenia and neutropenia with the combination of talazoparib and carboplatin was observed with HCC1806 and MDAMB231 (Fig. 5f–i).

No changes in haemoglobin (Hgb) levels were observed with treatment in the MX1 cohort (Fig. 5j). While the three combination approaches led to a mean reduction in Hgb levels of 36.2% in the HCC1806 cohort (Fig. 5h), the mean reduction was only 9.5% in the MDAMB231 cohort (Fig. 5l). While the moderate anaemia in HCC1806 could partly be considered an adverse event from talazoparib alone and partly due to a shorter recovery period post-completion of treatment, the mild anaemia with the MDAMB231 cohort is probably not “clinically” meaningful.

### Sequential combinations inhibit distant metastasis and migration, invasion

**Fig. 6:**
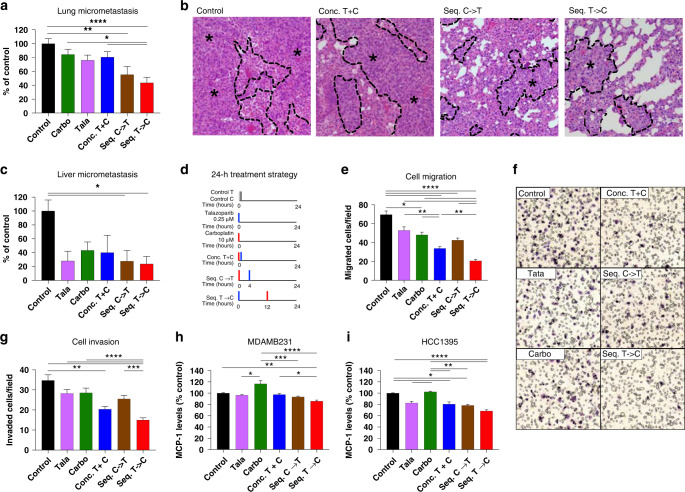
Sequential therapy inhibits distant metastasis in vivo, migration, invasion, and MCP-1 levels. Orthotopic xenograft of MDAMB231 was used to histologically evaluate (**a**, **b**) lung micrometastasis and (**c**) liver micrometastasis. **b** Representative images of H&E-stained sections of lung tissue, using ×20 objective. Metastatic cancer cells are demarcated from normal cells with a dashed black line and asterisks. The impact of different treatment strategies using a 24-h assay (**d**) in MDAMB231 cells on migration (**e**, **f**) and invasion (**g**) using transwell inserts + /− matrigel. **f** Representative images taken at 20x of MDAMB231 migrated cells that have been fixed and stained with hematoxylin. Secreted MCP-1 levels post treatment were quantified using an ELISA, in (**h**) MDAMB231 and (**i**) HCC1395 cells. Data are represented as mean +/– SEM. ANOVA with post test for multiple comparisons was performed. ****P < 0.0001; ***P < 0.001; **P < 0.01; *P < 0.05.

To further evaluate the impact of the combination of talazoparib and carboplatin on distant metastasis, we used an orthotopic xenograft model of MDAMB231, which is known for its high metastatic efficiency [33]. In comparison to control, both seq.C→T resulted in a 44.7% (*P* = 0.003) and seq.T→C led to a 56.4% (*P* < 0.0001) reduction in lung micrometastases, which was not observed with the concomitant combination (19.6% reduction, *P* = NS) (Fig. 6a, b). The seq.T→C approach also inhibited lung micrometastases by 40.8% in comparison to carboplatin (*P* = 0.01), and 36.9% in comparison to the concomitant approach (*P* < 0.05). Similar trends were also observed with liver micrometastasis (Fig. 6c). In comparison to control, seq. C→T resulted in a 72.3% (*P* = 0.03) reduction in micrometastasis, which was similar to the 76.3% (*P* = 0.02) reduction with the seq. T→C combination.

To further dissect the metastatic process, we evaluated the impact of talazoparib as monotherapy and in combination on cell migration and invasion in vitro (Fig. 6d–g). In MDAMB231 cells, as single agents, talazoparib and carboplatin resulted in a 24.0% (*P* = 0.05) and 30.8% (*P* = 0.01) reduction in cell migration, respectively. Once again, the seq.T→C demonstrated the most striking inhibition in cell migration (Fig. 6e, f), with 70.4% (*P* < 0.0001) reduction in comparison to control, 61.1% reduction (*P* < 0.0001) in comparison to talazoparib, and 39.0% reduction (*P* = 0.008) in comparison to conc.T + C. The other combination strategies also inhibited migration with 51.5% (*P* < 0.0001) reduction for the concomitant approach, and 38.9% (*P* < 0.0001) for the seq.C→T approach. The treatments as single agents or in combination demonstrated a similar trend with HS578T (Supplementary Fig. 8A). In comparison to control, seq.T→C also resulted in 56.8% (*P* < 0.0001) reduction in cell migration.

For cell invasion, in MDAMB231 cells, the seq.T→C approach also led to the strongest inhibition with 56.9% (*P* < 0.0001), 47.0% (*P* < 0.0001), 47.5% (*P* < 0.0001), and 41.3% (*P* = 0.0005) reduction in comparison to control, talazoparib, carboplatin, seq.C→T, and conc.T + C, respectively (Fig. 6g). The concomitant combination resulted in a 41.2% (*P* = 0.001) inhibition in cell invasion in comparison to control. In HS578T, the greatest inhibition was also demonstrated by seq.T→C with a 26.7% (*P* < 0.0001), 18.9% (*P* = 0.0002), and 21.3% (*P* < 0.0001) reduction in cell invasion in comparison to control, talazoparib, and carboplatin, respectively (Supplementary Fig. 8B). Conc.T + C demonstrated a 21.0% (*P* = 0.001) reduction in cell invasion, in comparison to control. Similar results were obtained with other PARPi. Olaparib or niraparib resulted in the greatest reduction in migration or invasion when administered witih the sequential PARPi-first combination approach (Supplementary Fig. 9).

Since PARPi have been shown to regulate cytokine signalling [39], we hypothesised that sequential talazoparib and carboplatin can enhance the inhibition of tumour secretion of chemokines which can explain its reduced migration capacity. We performed a chemokine array to profile 31 chemokines from MDAMB231 cell lysates (Supplementary Fig. 10A). In comparison to control, noteworthy reductions for seq. T→C were identified for CCL (C-C Motif Ligand) 2, CXCL (C-X-C Motif Ligand) 7, CCL18, CXCL9, CXCL1 and CCL7 (Supplementary Fig. 10B).

CCL2/MCP (Monocyte chemoattractant protein)-1 drives migration and invasion, and is implicated in cancer cell homing at the metastatic site [17, 39]. PARP1 knockdown was previously shown to significantly reduce levels of MCP-1 [39]. Interestingly, carboplatin upregulates MCP-1 [40], and blocking MCP-1 can improve the anti-tumour efficacy of carboplatin [41]. Therefore, it is plausible that pre-treatment with talazoparib can downregulate MCP-1 levels, enhancing the anti-migration potential of carboplatin. Hence, we further evaluated tumour secretion of MCP-1 in MDAMB231, and in a cell line known to express high levels of MCP-1, HCC1395 [42] (Fig. 6h, i). In MDAMB231, seq.T→C resulted in a 14.2% reduction in MCP-1 in comparison to control (*P* = 0.001), 30.7% reduction in comparison to carboplatin (*P* < 0.0001), and a 11.8% reduction in comparison to conc.T + C (*P* = 0.03). In HCC1395, seq.T→C also reduced MCP-1 levels by 31.5% in comparison to control (*P* < 0.0001), and by 33.9% in comparison to carboplatin (*P* < 0.0001). Conc.T + C also reduced MCP-1 levels by 19.5% (*P* = 0.049), and seq.C→T reduced MCP-1 levels by 21.7% (*P* = 0.01). Since the greatest downregulation of MCP-1 levels was demonstration by seq. T→C in comparison to carboplatin, this is suggestive that the sequential combination of talazoparib and carboplatin may be an important approach to inhibit MCP-1 levels that may otherwise be upregulated with carboplatin.

To better understand the underlying mechanisms for enhanced inhibition of metastasis, we also performed gene expression analysis of the metastatic lung tissue of the MDAMB231 xenograft to determine if there are differentially expressed pathways in the human cancer tissue between the treatment groups (Supplementary Figs. 11 and 12). We found that treatment groups clustered together, including talazoparib alone with carboplatin alone and control, while conc. T + C and seq. C→T also clustered together (Supplementary Fig. 11A). Principal component analysis showed the seq. T→C was distinct from the other combination groups. (Supplementary Fig. 11B). Pre-ranked gene set enrichment analysis (GSEA) demonstrated Seq.T→C had 9 significant gene sets that were downregulated in comparison to control, including pathways involved with the DNA damage response and metabolism, which were not identified with conc. T + C (Supplementary Fig. 11C–E). Furthermore, seq. T→C demonstrated downregulated gene sets in the DNA damage response including homologous recombination and mismatch repair, metabolism, and angiogenesis in comparison to conc. T + C and seq. C→T (Supplementary Fig. 12). Furthermore, in the mouse microenvironment, in comparison to control, seq. T→C demonstrated a downregulation of the CCR5 pathway, in which MCP-1 was a core-enriched gene (data not shown).

Altogether, the sequential administration of talazoparib and carboplatin resulted in a noteworthy reduction in distant metastasis, cell migration and invasion. Our results are suggestive that the PARPi-first combination may have a distinct mechanism of action in comparison to other combination approaches, which can significantly enhance the efficacy of each therapeutic agent.

## Discussion

Preclinical studies have commonly focused on the combination of PARPi and platinum-based therapy using BRCA^MUT^ models [43, 44]. To our knowledge, we are the first to evaluate the combination of a potent PARPi, talazoparib, with carboplatin in a large panel of TNBC cell lines. We identified synergy with the concurrent combination in 92.3% of TNBC cell lines comprising both BRCA^MUT^ and BRCA^WT^ subtypes. Talazoparib and carboplatin demonstrated a mean dose-reduction index of 2.8- and 3.7-fold, respectively. Similarly, sustained DNA damage and apoptosis responses were observed at lower drug concentrations of the combination, particularly in the PARPi-resistant cell lines. These results could partly explain the toxicity observed with the concomitant combination of talazoparib and carboplatin in a phase I trial, in which only a 0.25-fold reduction in talazoparib and carboplatin doses were utilised [28].

Sequential talazoparib and carboplatin was associated with a reduction in fork speed, DNA damage, and cell death in normal cells with, in comparison to TNBC cells. This is similar to what was previously reported: higher endogenous fork speeds correlated with lower endogenous replication stress in normal cells, which explained the reduced toxicity observed with the sequential PARPi combination approaches [30]. In our in vivo models, while the concomitant dosing tended to demonstrate the greatest toxicity (least weight gain), the sequential treatment approach had a different contextual impact. Whereas the least toxicity was observed with the carboplatin-first approach in the BRCA^MUT^ model, MX1, the least weight gain and haematologic toxicity was demonstrated by the talazoparib-first approach in the BRCA^WT^ model, MDAMB231. Since the same background strain was used, one plausible explanation for the differential toxicity could be different inflammatory responses induced by the BRCA^MUT^ versus BRCA^WT^ tumours [45, 46], which may in turn be modulated in different ways by the sequential administration of talazoparib and carboplatin. Therefore, our results are suggestive that a carboplatin-first approach in the BRCA^MUT^ context and talazoparib-first approach in the BRCA^WT^ context may offer patients the greatest safety profiles.

For our in vivo experiments, we based our dosing schedule of one dose of carboplatin and nine days of talazoparib on a xenograft model of subcutaneous tumour fragment implantation [34]. Our results showed that using orthotopic xenograft models with different tumour kinetics, that pausing the treatment for 4–16 days, allows for greater opportunity for the mice to regain weight and possibly mitigate anaemia, suggesting the importance of an intermittent dosing schedule. Indeed, clinical trials that have tested PARPi in combination have also shown improved efficacy and less toxicity with an intermittent dosing schedule [2]. For example, two trials dosed olaparib and veliparib on days 1–7 of 21-day cycles [47, 48]. Interestingly, the BROCADE3 trial also used a 2-day run in with veliparib for 7 days, and carboplatin administered on day 3 for a 21-day cycle. The combination of veliparib and carboplatin resulted in an improvement in PFS, with similar toxicity as chemotherapy [27].

While we understand some of the limitations of cells lines and their derived models [49], the strength of our study was the panel of TNBC cell lines, the use of three orthotopic xenograft models, including a BRCA^WT^ model with high metastatic efficiency. We showed comparable efficacy of the concurrent and sequential approaches for primary tumour inhibition. However, to our knowledge, we are the first to report that the sequential combination approaches strongly inhibit migration, invasion, and distant metastasis. The talazoparib-first combination demonstrated the greatest reduction in cell migration and invasion at 70.4% and 56.9%, respectively, with an enhanced effect in comparison to concurrent combination. In vivo, the talazoparib-first approach resulted in a 56.4% and 76.3% inhibition in lung and liver metastasis, respectively. Since we did not identify a differential impact on the tumour volumes with the sequential or concomitant combination approach, it is plausible that the impact upon migration and metastasis is independent of treatment effects on the primary tumour.

Furthermore, the talazoparib-first combination was associated with decreased expression of seven chemokines. The expression of one such chemokine, MCP-1, was downregulated the greatest with the talazoparib-first combination. Interestingly, chemotherapy including cisplatin has been shown to promote secretion of MCP-1, recruit inflammatory monocytes enriched with the receptor of MCP-1, CCR2, forming pre-metastatic niches, thus promoting distant metastasis in breast and lung cancer [50, 51]. Such chemotherapy-induced responses need to be countered immediately, prior to the development of distant metastasis. Since MCP-1 blockade can enhance carboplatin efficacy [41], it is plausible that pre-treatment with talazoparib decreases MCP-1 levels to enhance carboplatin sensitivity. However, since the magnitude of reduction of MCP-1 is less than the magnitude of migration reduction with different treatment combination approaches, it is likely that multiple factors may be contributing to inhibition of migration. Furthermore, stromal MCP-1 from the microenvironment was shown to more effective in reducing tumour burden than blockade of MCP-1 induced by the proper tumour itself [41]. While we did identify a downregulation of the MCP-1 pathway in the microenvironment, the therapeutic impact on the microenvironment needs to be further explored.

Finally, we evaluated the pathways that were differentially expressed by the different treatment groups using metastatic lung tissue. We found that DNA damage response pathways were distinctly downregulated with seq.T→C in comparison to control and other combination groups. We and others have previously shown that the downregulation of these pathways allow for enhanced sensitivity to PARPi [22, 52], suggesting that the sequential approach is enhancing PARPi sensitivity, particularly in this BRCA^WT^ model.

Taken together, we have provided a comprehensive preclinical analysis of the combination of talazoparib and carboplatin in TNBC. We have demonstrated that the combination is synergistic in most TNBC cell lines. While the sequence of administration does not impact tumour proliferation or tumour growth, sequential administration of talazoparib and carboplatin can decrease toxicity and significantly inhibit migration, invasion, and distant metastasis. Therefore, our results lead the way to future clinical trials with the evaluation of the sequential combination of talazoparib and carboplatin in early breast cancer, potentially providing an effective approach to eradicate micrometastatic disease in TNBC patients.

## Supplementary information

Supplementary Methods

Supplementary Figures

Supplementary Table 1

ARRIVE Checklist

Reporting Summary

## Data Availability

All data and materials are available upon request.
