# Supplementary material for: Sequential targeting of PARP with carboplatin inhibits primary tumour growth and distant metastasis in triple-negative breast cancer
Source: Br J Cancer. 2023 Mar 20;128(10):1964–75. doi: 10.1038/s41416-023-02226-w (PMC10147920; doi:10.1038/s41416-023-02226-w)
Supplement: Supplementary file 1 — Supplementary Methods [file 41416_2023_2226_MOESM1_ESM.pdf]

Supplementary Methods

REAGENT or RESOURCE	SOURCE	IDENTIFIER
**Antibodies**		
Cleaved-PARP	Cell Signaling	Cat #9546
53BP1	Novus Biologicals	Cat #NB100-904
Alexa 647 donkey anti-rabbit	Life Technologies	Cat #A31573
Alexa 488 donkey anti-mouse	Life Technologies	Cat #A21202
HCS Nuclear Mask	Life Technologies	Cat #H10325
gH2AX-AlexaFluor 647	BioLegend	Cat #613408
Phospho-Histone H3-AlexaFluor 488 (Ser-10)	Cell Signaling	Cat #3465
Cleaved-caspase 3-Pacific Blue	Cell Signaling	Cat #8788
Rat anti-BrdU (clone BU1/75 (ICR1))	Abcam	Cat #ab6326
Mouse anti-BrdU [clone B44]	BD Biosciences	Cat #347580
Anti-Rat AlexaFluor 568	Invitrogen	Cat #A11077
**Chemicals, peptides, and recombinant proteins**		
Talazoparib	Pfizer	
Carboplatin (in-vitro)	Selleckchem	Cat #S1215
Carboplatin (in-vivo)	CHUM pharmacy	
Olaparib	Selleckchem	Cat #S1060
Niraparib	Selleckchem	Cat #S2741
Hematoxylin QS Counterstain	Vector Laboratories	Cat #H3404
Propidium Iodide	Invitrogen	Cat #P3566
Chloro-2'-deoxyuridine (CldU)	Sigma-Aldrich	Cat #C6891
5-Iodo-2'-deoxyuridine (IdU)	Sigma-Aldrich	Cat #I7125
**Critical commercial assays**		
Cell lysis buffer	Cell Signalling	Cat #9803
Proteome Profiler Array – Human Chemokine Array Kit	R&D Systems	Cat #ARY017
Quantikine ELISA – Human CCL2-MCP1 Immunoassay	R&D Systems	Cat #SCP00
Matrigel Matrix Phenol Red Free	Fisher Scientific	Cat #CB40234C
Collagen Type 1, rat tail	Millipore Sigma	Cat #08-115
Cultrex Reduced Growth Factor Basement Membrane Extract	R&D Systems	Cat. #3433-005-01
ProLong™ Gold antifade Mountant	Invitrogen	Cat #P36930
Pierce BCA Protein Assay Kit	ThermoFisher	Cat #23225
Rneasy FFPE kit	Qiagen	Cat #73504
**Experimental models: Cell lines**		
MDAMB231	JW Gray Lab	N/A
HS578T	JW Gray Lab	N/A
SUM149PT	JW Gray Lab	N/A
HCC1395	JW Gray Lab	N/A
HCC1806	JW Gray Lab	N/A
HCC1937	JW Gray Lab	N/A
MX1	JW Gray Lab	N/A
HCC2185	JW Gray Lab	N/A
HCC38	JW Gray Lab	N/A
HCC1143	JW Gray Lab	N/A
MDAMB436	ATCC	Cat #HTB-130
BT20	ATCC	Cat #HTB-19
BT549	ATCC	Cat #HTB-122
HCC1187	ATCC	Cat #CRL-2322
BM-MSC	ATCC	PCS #500-012
MCF10A	AddexBio	Cat #C0006015
**Experimental models: Mice**		
NOD.Cg-Prkdcscid Il2rgtm1Wjl/SzJ	The Jackson Laboratory	Cat# 005557
**Software and algorithms**		
GraphPad PRISM 8	GraphPad software	https://www.graphpad.com/scientific-software/prism/
STATA SE, Version 15	StataCorp LLC	https://www.stata.com/
FlowJo	BD Biosciences	https://www.flowjo.com/
Compusyn	ComboSyn Incorporated	https://www.combosyn.com
ImageJ	NIH	https://imagej.nih.gov/ij/
Harmony High Content Imaging and Analysis Software	Perkin Elmer	https://www.perkinelmer.com/product/harmony-4-8-office-hh17000001
GenePattern, Version 3.9.11	National Cancer Institute's Informatics Technology for Cancer Research program and the National Institute of General Medical Sciences	https://cloud.genepattern.org/gp/pages/index.jsf

10-day chemosensitivity assay and immunofluorescence staining, analysis and visualization was performed as previously described (1) and detailed below**.**

***In-vitro* 10-day chemosensitivity assay**

For each cell line, cells were seeded in 96 well plates (Cat #3603, Corning) in triplicate wells. Cells were treated with either 9 concentrations (5-fold dilutions) of talazoparib (Pfizer) wherein the highest concentration was 5 μM, or 9 concentrations (3-fold dilutions) of carboplatin (Cat #S1215, Selleckchem), wherein the highest concentration was 100 μM, or the combination of talazoparib and carboplatin with the same 9 concentrations. Drug treatment occurred 24 hours after cell seeding. Media and drug were changed after 4 to 5 days. Cells were treated for a total of 9 days and then were fixed and permeabilized with 4% paraformaldehyde, diluted from stock paraformaldehyde 32% solution, EM grade (Cat #15714, Electron Microscopy Sciences), and 0.3% Triton X-100 (Cat #T9284, Sigma-Aldrich).

Immunofluorescence staining

We prepared a primary and secondary antibody solution using 2% BSA (Cat #001-000-162, Jackson ImmunoResearch). We used the following primary antibodies: cl-PARP (1:200, Cat #9546, Cell Signaling Technology) and 53BP1 antibody (1:500, Cat #NB100-904, Novus Biologicals). Secondary antibodies included Alexa 488 donkey anti-mouse (1:300, cat # A21202, Life Technologies) and Alexa 647 donkey anti-rabbit (1:300, Cat #A31573, Life Technologies). We used HCS Nuclear Mask (1:2000, Cat #H10325, Life Technologies) to stain the nucleus, which was added at the time of the secondary antibody solution.

IC50 Calculation

Using the drug sensitivity assay plates treated with carboplatin or talazoparib, nuclear stained viable cell counts were obtained from Operetta Analysis Software. Drug dose response curves were calculated from which IC50 values were derived using Graph Pad Prism 8 software. IC50 values were obtained from 2-5 replicate assays.

Statistical analysis for immunofluorescence

We quantified the mean number of 53BP1 foci per nucleus, percentage of cells positive for 53BP1 foci formation, and percentage of cl-PARP+ cells. The 53BP1 product score was calculated by taking the product of the mean number of 53BP1 foci and percentage of cells positive for 53BP1 for each drug concentration. All single-cell analysis was performed using STATA SE (version 15.1, StataCorp).

Data visualization of immunofluorescence

For each drug concentration of talazoparib, carboplatin, and the combination of talazoparib and carboplatin, we created two heatmaps to visualize the 53BP1 product score and the percentage of cells positive for cl-PARP. In all cases, data from each drug treatment were normalized to the DMSO control. Values were scaled to the maximum value to compare across cell lines. Heatmaps were created using a double gradient colour scheme in GraphPad Prism 8.

10-day chemosensitivity assay to evaluate impact of different combination strategies

For the 10-day proliferation experiment described in Fig. 3, the same assay was used as described above, in which image-based analysis was used to enumerate cell counts on day 10. Cells were treated as follows: either 0.3% DMSO control, talazoparib or carboplatin on day 1 if monotherapy, talazoparib + carboplatin on day 1 as concomitant combination (conc. T+C), carboplatin on day 1 followed by talazoparib 24 hours (h) later (seq. C->T), or talazoparib administered on day 1 followed by talazoparib plus carboplatin 48h later (seq. T->C) to model the *in-vivo* treatment strategy with a talazoparib run-in.

Drug Treatment Strategies for 72h and 24h assays

For DNA fiber and flow cytometry experiments, cells were treated for 72h. Cells either received DMSO control, talazoparib at 0.25 μM, carboplatin at 10 μM, concurrent combination of talazoparib at 0.25 μM plus carboplatin at 10 μM. Sequential combination was administered either with carboplatin at 10 μM first for 24h, and then talazoparib for 48h (seq. C->T) or with a talazoparib run-in, in which talazoparib was first administered

at 0.25 μM for 48 hours, followed by carboplatin (10 μM) plus talazoparib (0.25 μM) (seq. T->C).

For the cell migration, invasion, chemokine array and ELISA experiments, cells were treated for 24h. To model the in-vivo dosing, cells were treated with either talazoparib at 0.25 μM, carboplatin at 10μM, the concurrent combination of talazoparib plus carboplatin (conc. T+C), the sequential combination of carboplatin first for 4h, followed by talazoparib for 20h (seq. C->T), or the sequential combination of talazoparib first for 12 hours, followed by carboplatin for 12 hours (seq. T->C). Following drug treatment, cells were starved for 24 hours.

Flow Cytometry Gating Strategy

Flow Cytometry Gating Strategy
MDAMB231 cells treated with carboplatin for the detection of pH3+gH2AX+ cells.

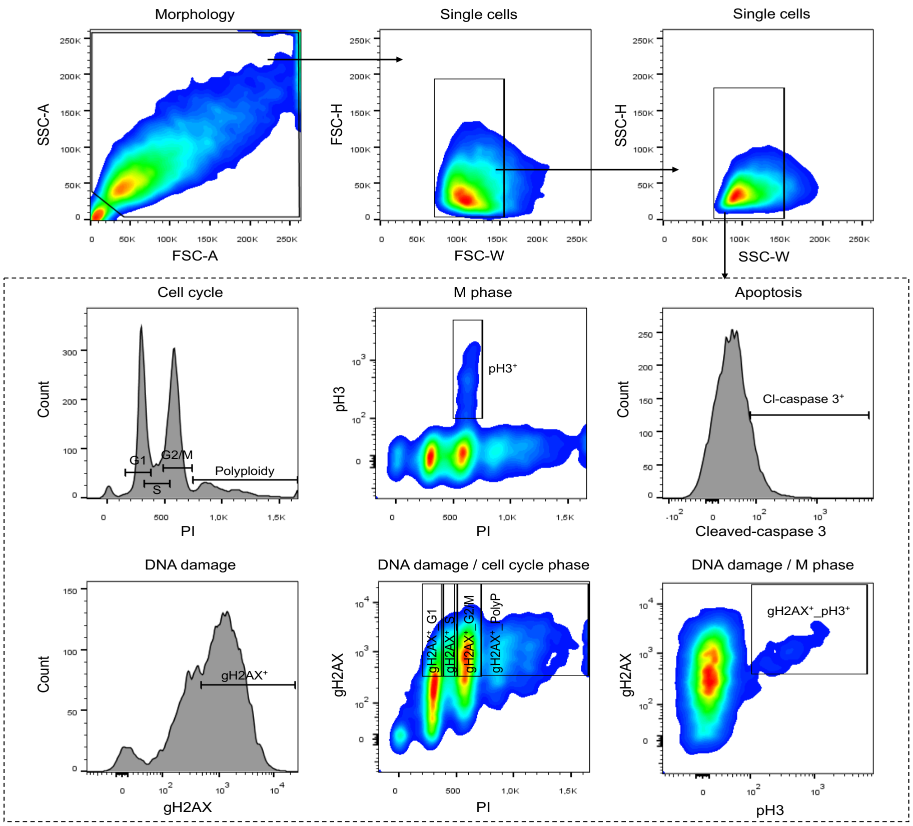

Animal Studies

Pilot Experiments

NOD.Cg-Prkdcscid Il2rgtm1Wjl/SzJ (Stock #005557, Jackson Laboratory) were used for all experiments. Mice were acclimatized at our institutional animal quarters (Centre de Recherche de Centre Hospitalier de l'Université de Montréal, (CRCHUM), for 2 weeks prior to intervention. Orthotopic implantation of tumors was performed to better understand the context of the microenvironment and to increase metastatic propensity. For the three orthotopic xenograft models, pilot studies were first conducted at 2 or 5 million cells that were implanted in one mammary fat pad for each mouse (2). Orthotopic surgical implantation consisted of general anesthesia, followed by an incision, in which 0.2 mL of cells were injected into the mammary fat pad #4 under direct vision. Here, tumor volumes and signs of animal distress were recorded, and the date of endpoint for necropsy was identified for subsequent treatment trials. The presence of significant liver and lung micrometastases was only identified in the MDAMB231 orthotopic xenograft model (in 4/4 evaluated mice).

Treatment Experiments

Tumors were measured with digital calipers and mice weighed twice weekly. Tumor volumes were calculated using the formula $(x^2 * y)/2$, where x and y represent the shortest and longest diameters, respectively.

Carboplatin (Centre Hospitalier de l'Université de Montréal (CHUM) pharmacy) was administered intraperitoneally (i.p.) at 35 mg/kg and talazoparib was administered by oral gavage (o.g.) at 0.33 mg/kg. Vehicles used were normal saline for carboplatin, and 5% polyethylene glycol 400 for talazoparib. For each xenograft model, mice either received vehicle control, talazoparib alone (days 1-9), carboplatin alone on day 1, concomitant treatment with talazoparib (day 1-9) and carboplatin on day 1 (conc. T+C); sequential combination with carboplatin first on day 1 followed by talazoparib on day 2-10 (seq. C->T); and another sequential with a talazoparib run-in (days 1-2) followed by carboplatin on day 3, continued with talazoparib (days 3-9).

Due to different tumor kinetics, for HCC1806, the necropsy was performed on day 22 (4 days after completion of drug treatment), and for MDAMB231 and MX1, necropsy was performed on day 42 (16 days after end of treatments). One mouse in the MDAMB231 cohort which received concomitant talazoparib and carboplatin which had to be sacrificed early due to toxicity was excluded from the final *ex-vivo* tumor volume or lung metastasis evaluation.

At necropsy, plasma was procured via a terminal cardiac puncture and processed for a complete blood count at the CHUM Hematology Laboratory. Any coagulated samples were excluded from analysis. Some blood samples were missing manual differentiation, for which neutrophil counts could not be obtained. For each mouse, tumor, lung, and liver organs were harvested. *Ex-vivo* tumors at necropsy were measured by two people (T.H. and S.H.). Lung and liver were examined for macroscopic disease and then fixed in 10% formaldehyde, and formalin-fixed paraffin-embedded blocks were created. Pathologist, D.T-T. blindly reviewed the entire slide of liver and lung parenchyma and reported percentage of area occupied by cancer cells.

RNA-Seq Gene Expression and Analysis

We selected 5 representative archived FFPE tissue blocks from each treatment group from metastatic lung tissue from the MDAMB231 orthotopic xenograft. RNA was extracted using RNeasy FFPE kit (Cat # 73504) at the Molecular Pathology Platform (CRCHUM). RNA was quantified using Qubit (Thermo Scientific) and quality was assessed with the 2100 Bioanalyzer (Agilent Technologies). Samples with a DV200 > 30% were selected. Transcriptome libraries were generated using QIAseq FastSelect ribodepletion selection (Qiagen), followed by KAPA RNA HyperPrep (Roche) at the Institute for Research in Immunology and Cancer (IRIC, Montreal). Sequencing was performed on the Illumina NovaSeq6000 (PE100), at the McGill Genome Center, Montreal. 120M Paired-End reads were obtained per sample.

The first part of the analysis including normalization and DeSEq2 analysis was performed at the Bioinformatics Facility at IRIC. Sequences were trimmed for sequencing adapters and low quality 3' bases using Trimmomatic version 0.35 (3) and aligned on a hybrid

genome formed of the reference human genome version GRCh38 (gene annotation from Gencode version 37, based on Ensembl 103) and mouse genome version GRCm38 (Gencode version M25) using STAR version 2.7.1a (4). DESeq2 version 1.30.1 (5) was then used to normalize gene readcounts (*from the human genes only*) and produce the sample clustering. A principal component analysis was also performed to display the first two most significant components. Using the log2-fold change values from the DESeq2 analysis, all expressed genes were ranked and a pre-ranked Gene Set Enrichment Analysis was performed using GenePattern (6). Gene sets from MsigDB were used, including Hallmarks and KEGG for human tissue, and Hallmarks and Biocarta for mouse tissue. FDR cutoff of 25% was used to identify statistically significant gene sets.

References

1. Hassan S, Esch A, Liby T, Gray JW, Heiser LM. Pathway-Enriched Gene Signature Associated with 53BP1 Response to PARP Inhibition in Triple-Negative Breast Cancer. Molecular cancer therapeutics. 2017;16(12):2892-901.
2. Iorns E, Drews-Elger K, Ward TM, Dean S, Clarke J, Berry D, et al. A new mouse model for the study of human breast cancer metastasis. PloS one. 2012;7(10):e47995.
3. Bolger, A. M., Lohse, M., & Usadel, B. Trimmomatic: A flexible trimmer for Illumina Sequence Data. Bioinformatics, 2014. btu170.
4. Dobin A1, Davis CA, Schlesinger F, Drenkow J, Zaleski C, Jha S, Batut P, Chaisson M, Gingeras TR. STAR: ultrafast universal RNA-seq aligner. Bioinformatics. 2013 Jan 1;29(1):15-21.
5. Love MI, Huber W and Anders S. Moderated estimation of fold change and dispersion for RNA-seq data with DESeq2. Genome Biology15, 2014: pp. 550.
6. Reich M, Liefeld T, Gould J, Lerner J, Tamayo P, Mesirov JP. GenePattern 2.0 Nature Genetics 38 no. 5, 2006: pp500-501.
