# Supplementary material for: Sequential targeting of PARP with carboplatin inhibits primary tumour growth and distant metastasis in triple-negative breast cancer
Source: Br J Cancer. 2023 Mar 20;128(10):1964–75. doi: 10.1038/s41416-023-02226-w (PMC10147920; doi:10.1038/s41416-023-02226-w)
Supplement: Supplementary file 2 — Supplementary Figures [file 41416_2023_2226_MOESM2_ESM.pdf]

## Supplementary Figure 1

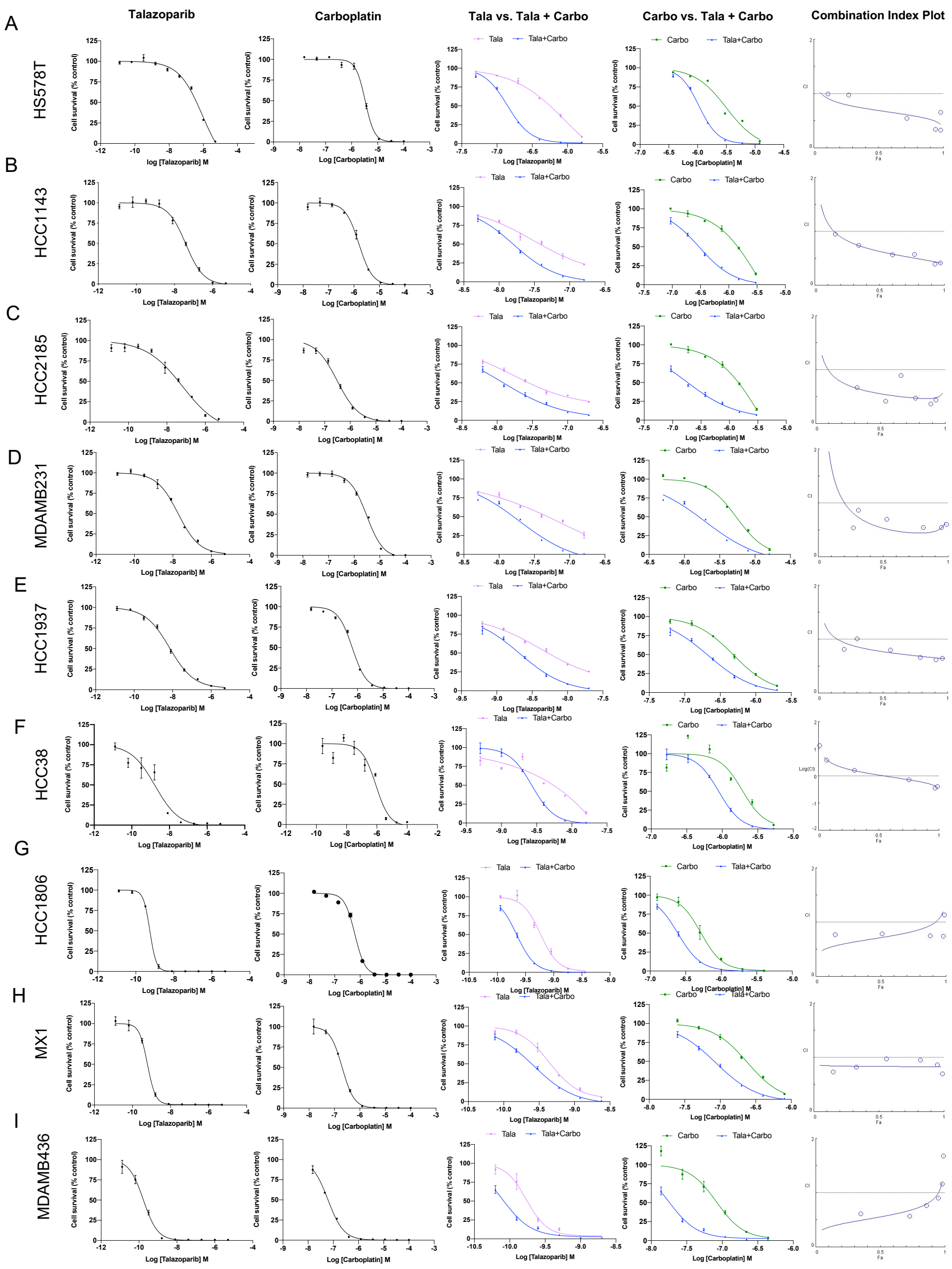


**Supplementary Fig. 1**. **Dose-response curves of talazoparib, carboplatin, and combination**. Representative IC50 dose-response curves of single-agent and combination effect from nine cell lines including A) HS578T; B) HCC1143; C) HCC2185; D) MDAMB231; E) HCC1937; F) HCC38; G) HCC1806; H) MX1; and I) MDAMB436. Dose-response curves are in the first panel from the extreme left with nine concentrations of talazoparib from 0.013 nM to 5 μM with 1/5 dilutions, and in the second column, with carboplatin from 0.015 to 100 μM with 1/3 dilutions. In the third and fourth columns are comparative dose-response curves with talazoparib compared to combination of talazoparib plus carboplatin, and carboplatin compared to talazoparib plus carboplatin, respectively. In the comparative combination drug dose-response curves, six concentrations centered on the single-agent IC50 values were used, with 1/2 dilutions *(n = 2-5)*. Points are mean values with SEM bars. In the last column on the right are combination index plots, with combination index along the y-axis, and Fa values along the x-axis.

**Supplementary Figure 2**

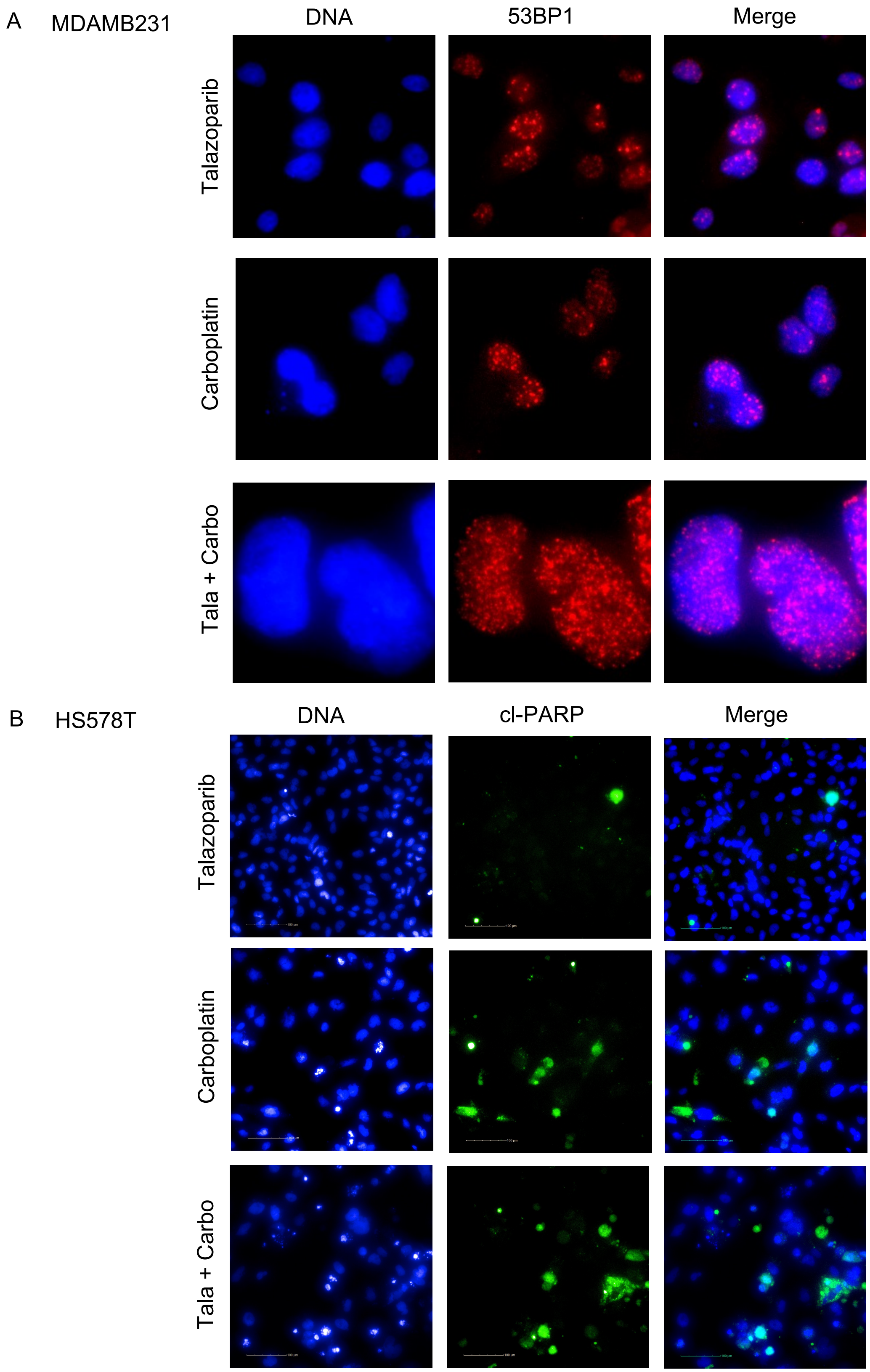


**Supplementary Fig. 2**. Representative single channel images of A) 53BP1 foci in MDAMB231 cells and B) cleaved (cl)-PARP expression in HS578T cell. Left column shows nuclei stained with HCS nuclear mask, middle column indicates 53BP1 foci/cl-PARP expression, and right column are the merged images.

**Supplementary Figure 3**

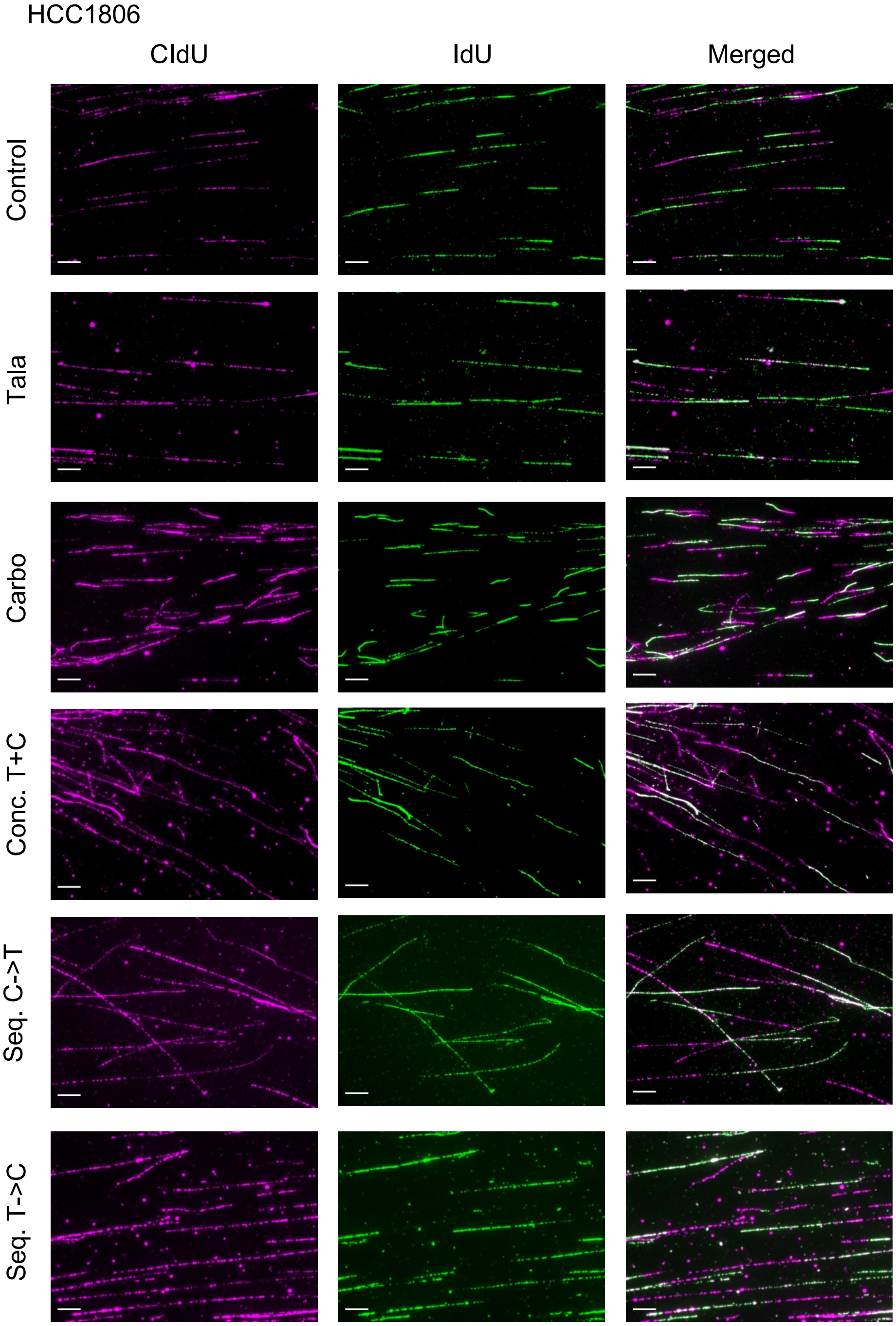


**Supplementary Fig. 3. DNA fiber images of HCC1806 cells treated with talazoparib or carboplatin as single-agents or in combination.** Representative single-channel images of CldU in magenta (left), IdU in green (middle), and merged images (right). Scale bars refer to 10 microns.

**Supplementary Figure 4**

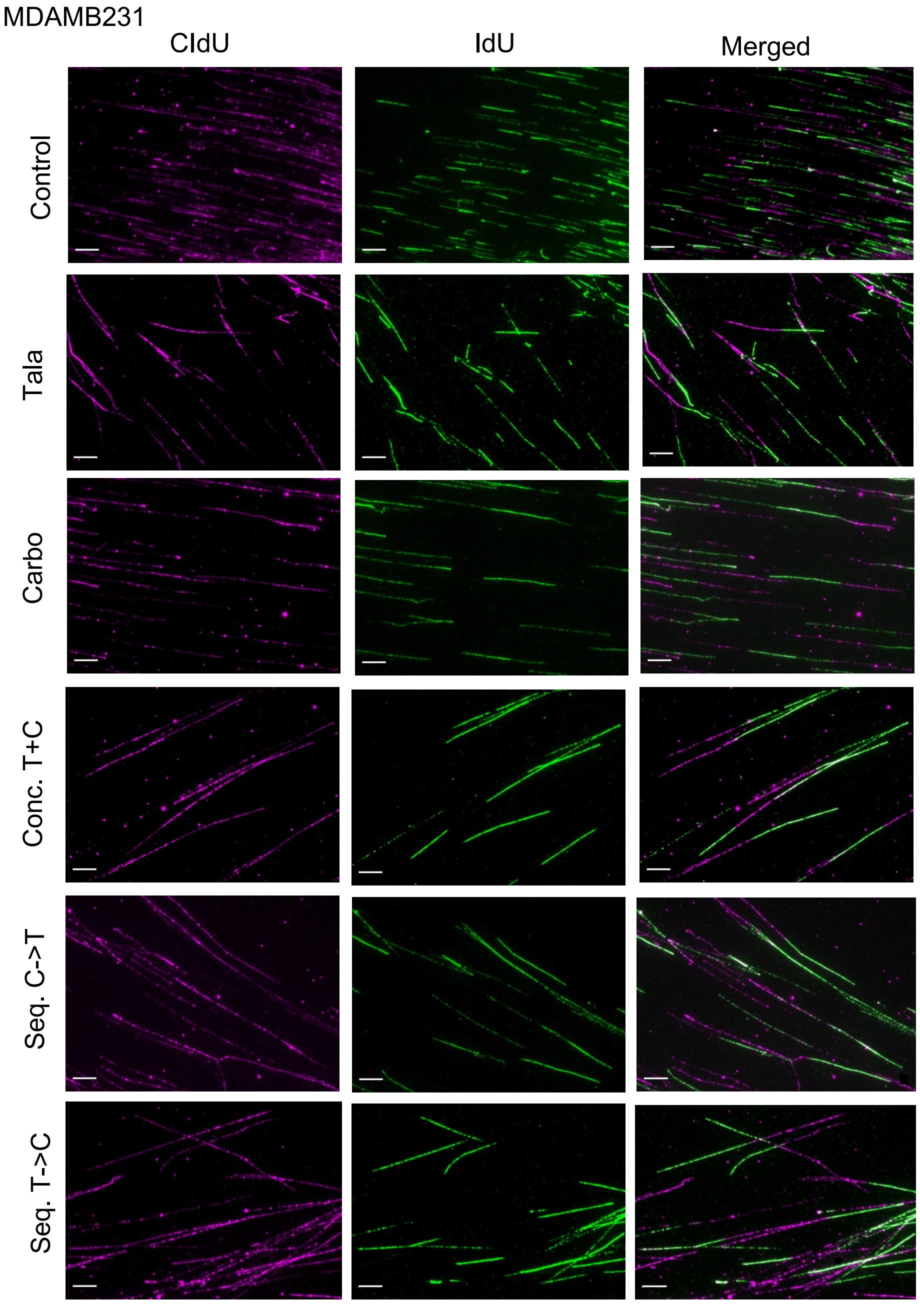


**Supplementary Fig. 4. DNA fiber images of MDAMB231 cells treated with talazoparib or carboplatin as single-agents or in combination.** Representative single-channel images of CldU in magenta (left), IdU in green (middle), and merged images (right). Scale bars refer to 10 microns.

**Supplementary Figure 5**

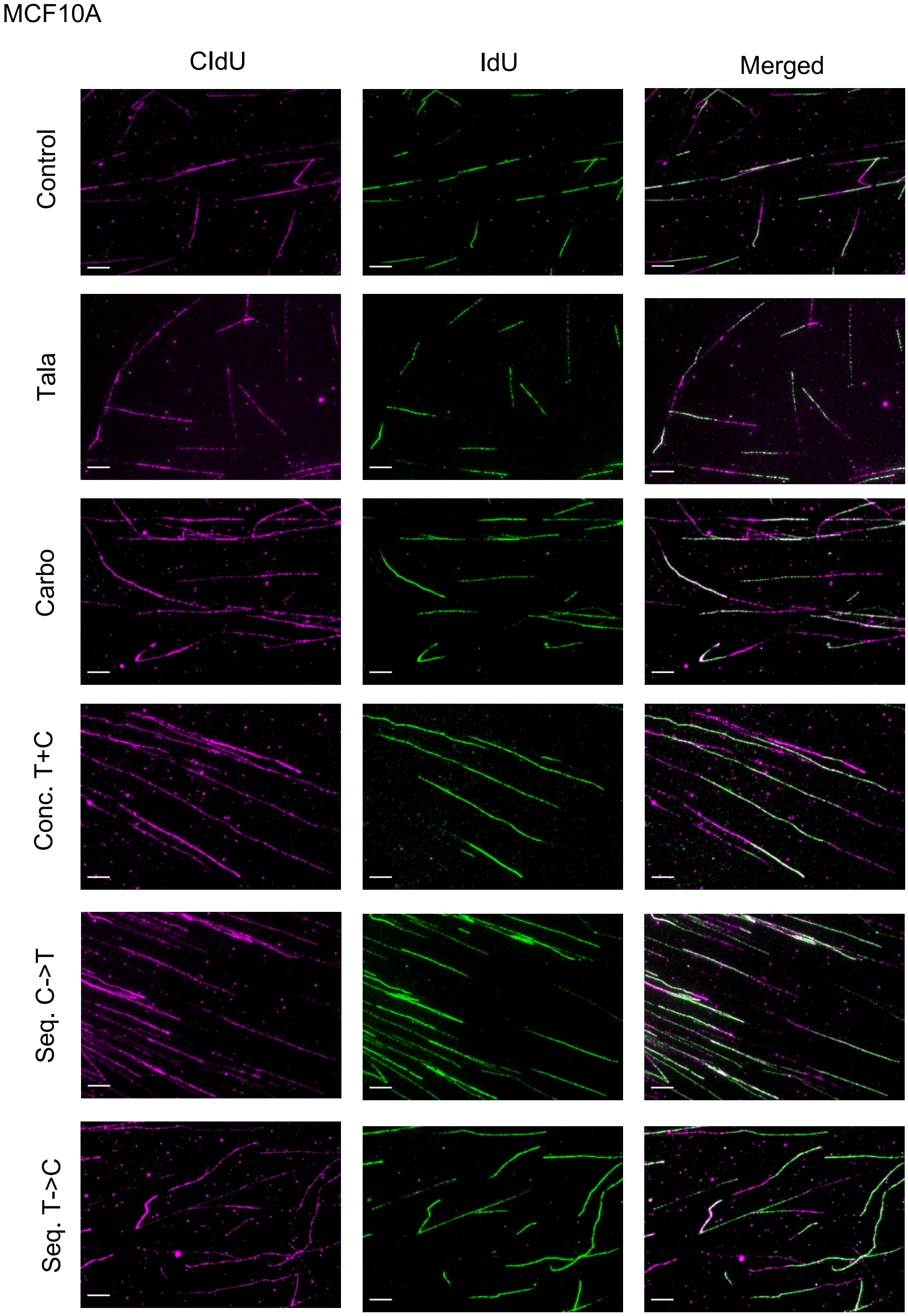


**Supplementary Fig. 5. DNA fiber images of MCF10A cells treated with talazoparib or carboplatin as single-agents or in combination.** Representative single-channel images of CldU in magenta (left), IdU in green (middle), and merged images (right). Scale bars refer to 10 microns.

**Supplementary Figure 6**

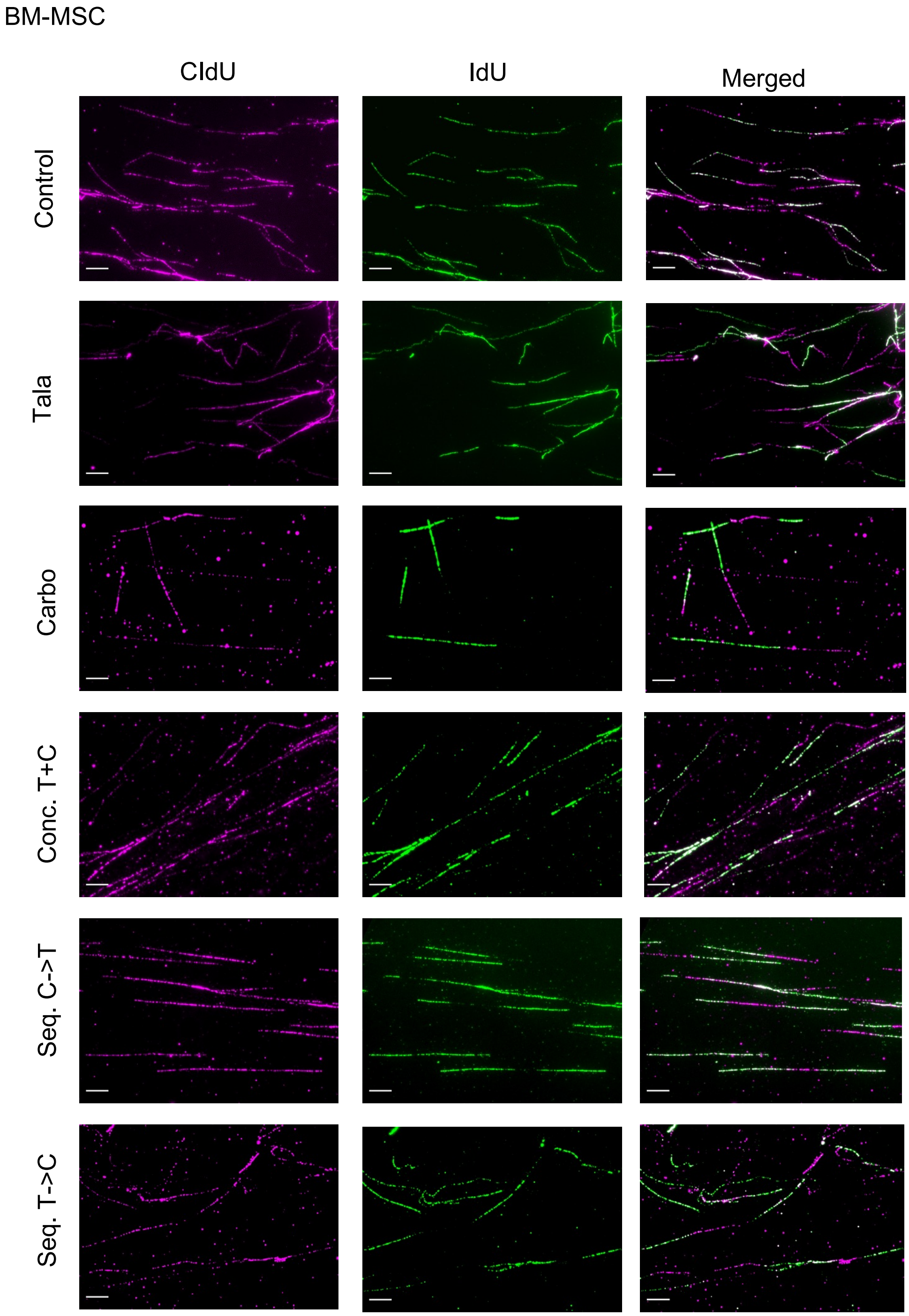


**Supplementary Fig. 6. DNA fiber images of BM-MSC cells treated with talazoparib or carboplatin as single-agents or in combination.** Representative single-channel images of CldU in magenta (left), IdU in green (middle), and merged images (right). Scale bars refer to 10 microns.

**Supplementary Figure 7**

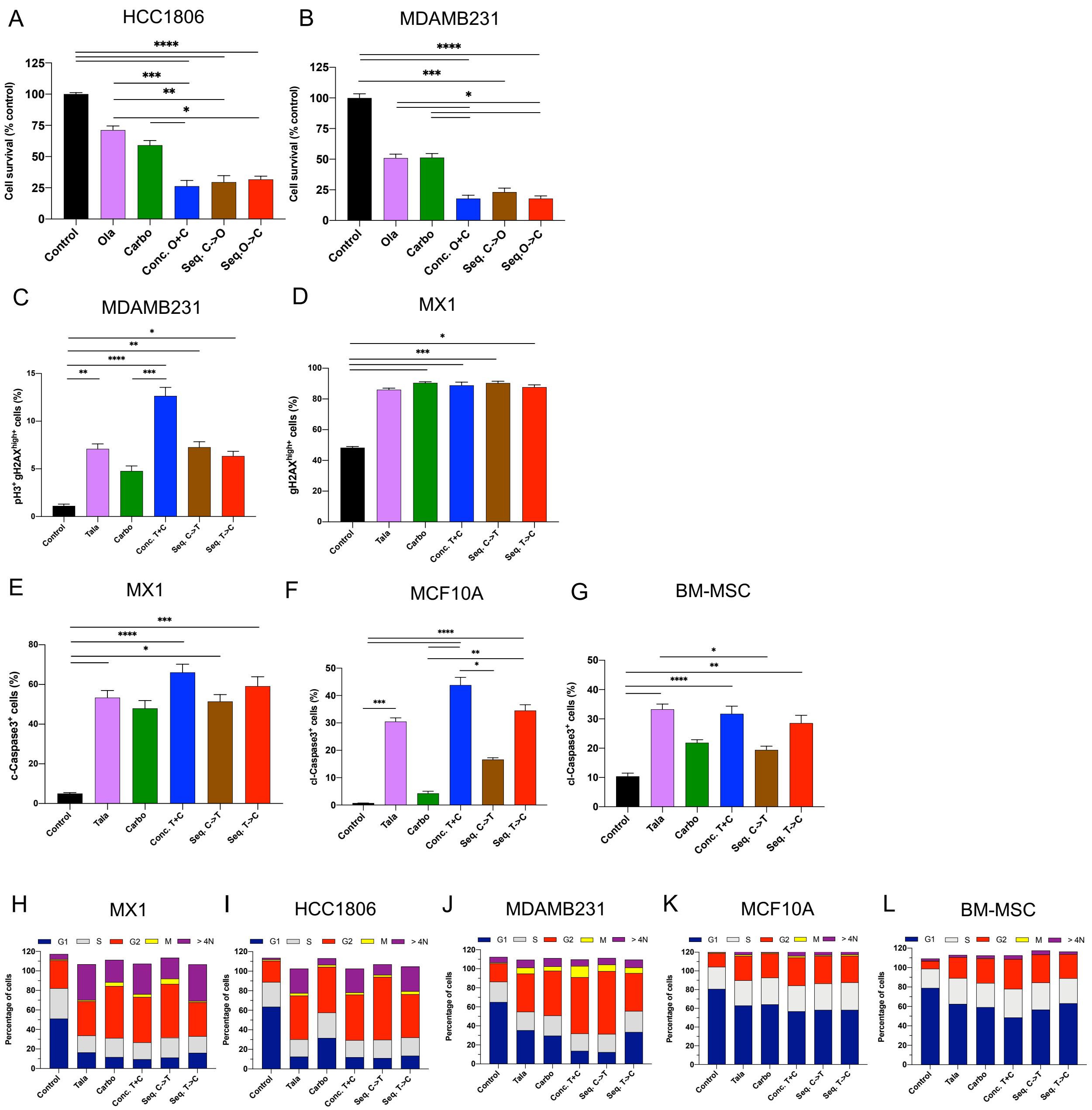


**Supplementary Fig. 7. Impact of treatments on cell proliferation, DNA damage, apoptosis and cell cycle changes** *(n = 3-4)*. Impact of concurrent or sequential combination of olaparib and carboplatin on cell proliferation using a 9-day treatment assay in (A) HCC1806, and (B) MDAMB231 cell lines. The impact of talazoparib and carboplatin was assessed using 72-hour assays on DNA damage (C,D), apoptosis (E-G), and cell cycle changes (H-L). DNA damage during mitosis is shown in MDAMB231 cells, represented by pH3+ γ-H2AX+ cells in (C) and total γ-H2AX+ cells in MX1 is shown in (D). Apoptosis with cleaved (cl)-caspase 3+ cells are shown in MX1 (a p53-wild type TNBC cell line, since chemotherapy-treated p53-mutant cancer cells can be resistant to apoptosis (Shah and Schwartz, 2001), MCF10A, and BM-MSC cells in (E-G). Impact of treatment on cell cycle changes are shown in MX1, HCC1806, MDAMB231, MCF10A, and BM-MSC (H-L).

**Reference:**


Shah, M.A., and Schwartz, G.K. (2001). Cell cycle-mediated drug resistance: an emerging concept in cancer therapy. Clinical cancer research, *7*, 2168-2181.

**Supplementary Figure 8**

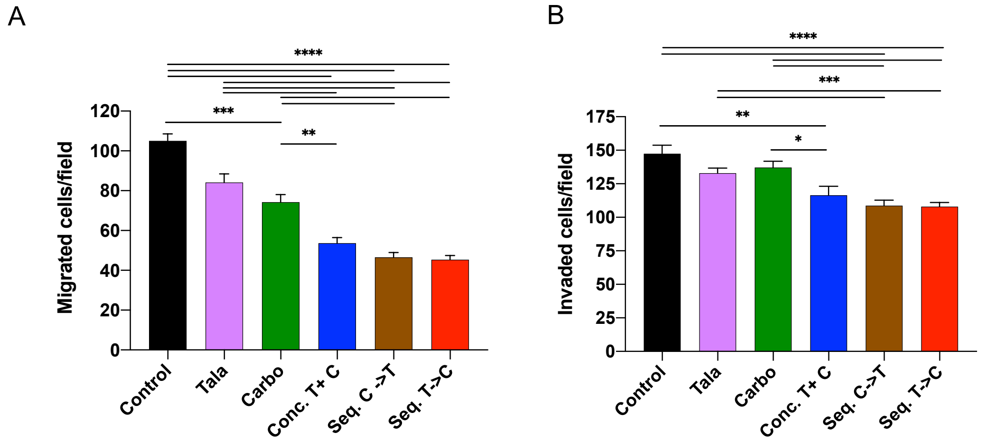


**Supplementary Fig. 8. Cell migration and invasion in HS578T cells.** A) Cell migration assay in which mean migrated cells/field are along the y-axis and different treatments along the x-axis. B) Invasion assay in which cells that invaded through the transwell membrane with matrigel were quantified along the y-axis, and different treatments along the x-axis. Six images at 20X objective were used to count cells. Data are represented as mean +/- SEM. Kruskal-Wallis tests with Dunn's post-test was performed. **** P< 0.0001; *** P<0.001; **P<0.01; *P<0.05 *(n = 3).*

**Supplementary Figure 9**

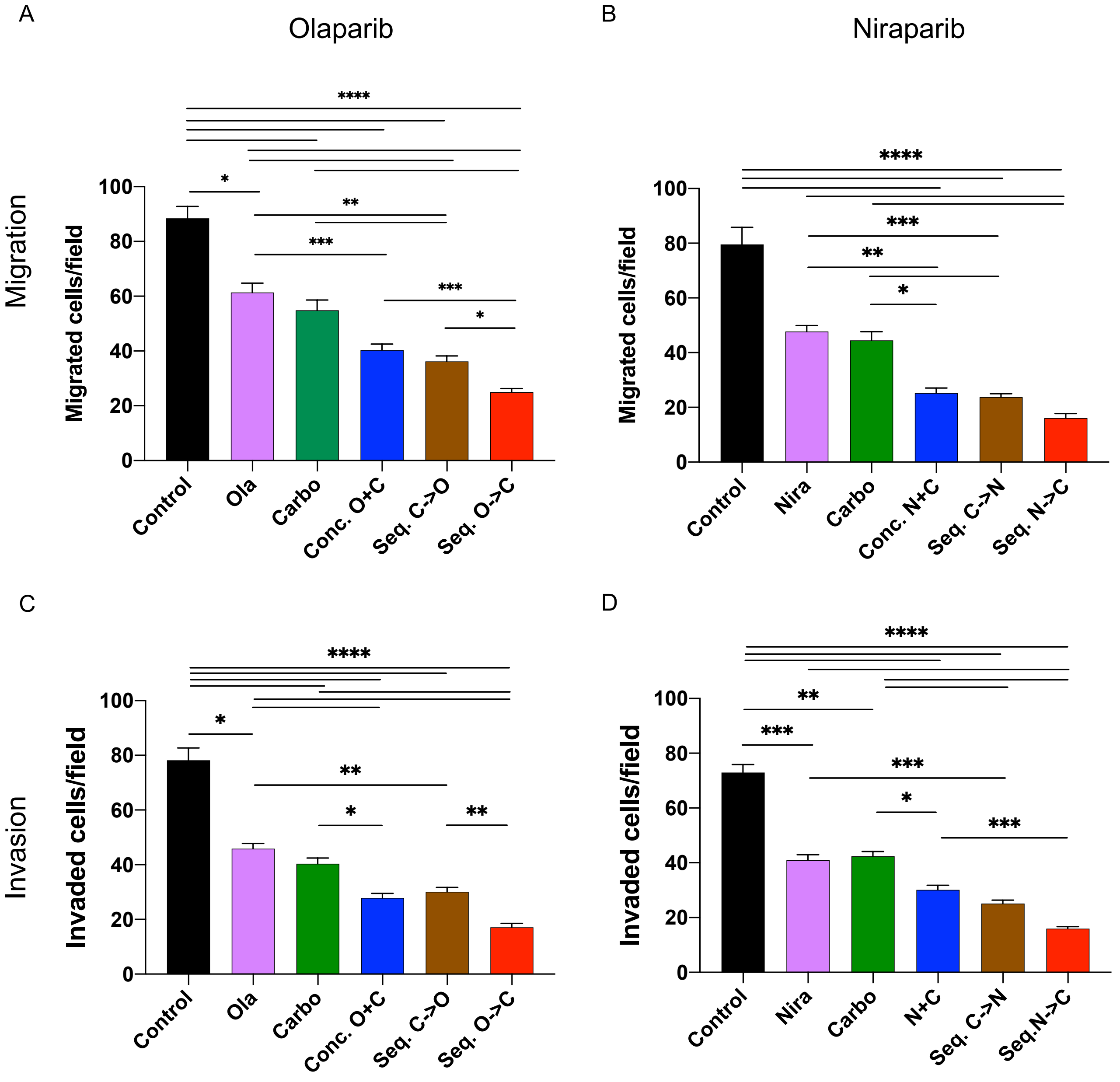


**Supplementary Fig. 9. Cell migration and invasion using additional PARP inhibitors, olaparib on the top panel, and niraparib on the bottom panel.** A,B) Cell migration assay in which mean migrated cells/field are along the y-axis and different treatments along the x-axis. C,D) Invasion assay in which cells that invaded through the transwell membrane with matrigel were quantified along the y-axis, and different treatments along the x-axis. Six images at 20X objective were used to count cells. Data are represented as mean +/- SEM. Kruskal-Wallis tests with Dunn's post-test was performed. **** P< 0.0001; *** P<0.001; **P<0.01; *P<0.05 *(n = 2-3).*

**Supplementary Figure 10**

A

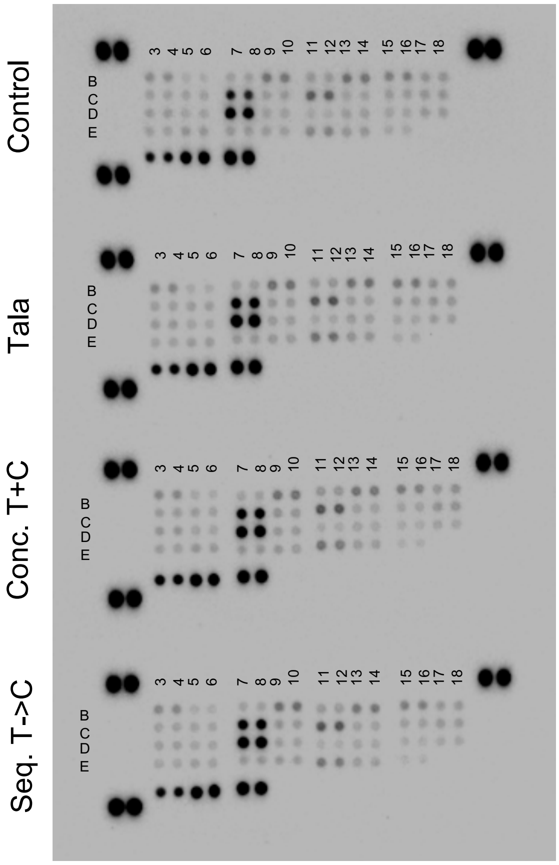


C17,18; MCP-1
E3, E4; NAP-2
E5, E6; PARC
D9, D10; MIG
B17, B18; GRO-alpha
E11, E12: SDF-1
D3, D4: MCP-3

B

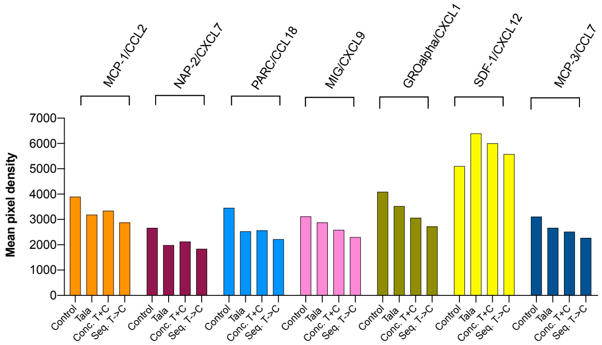


**Supplementary Fig. 10. Chemokine array of MDAMB231 cell lysates.** A) MDAMB231 cells treated with control (top panel), talazoparib (2nd panel), concomitant talazoparib + carboplatin (conc. T+C) (3rd panel), and sequential talazoparib-> carboplatin (seq. T->C) (4th/bottom panel). Cell lysates were then used with the chemokine array kit (Cat. ARY017, R&D Systems) to detect differences in 31 chemokines, including: 6Ckine, CCL28, CXCL16, Chemerin, ENA-78, Eotaxn-3, Fractalkine, GRO-alpha. HCC-1, I-309, IL-8, IL-16. IP-10, I-TAC, Lymphotactin, MCP-1, MCP-3, MDC, Midkine, MIG, MIP-1α/β, MIP-1δ, MIP-3α, MIP-3β, NAP-2, PARC, PF4, RANTES, SDF-1, TARC, VCC-1. B) Selected chemokines showing differential expression from control and talazoparib-first combination approach using a chemokine array with MDAMB231 cell lysates. The x-axis are different treatment strategies, and the y-axis are spots that have been quantified using the QuickSpots (Western vision) software.

## Supplementary Figure 11

A

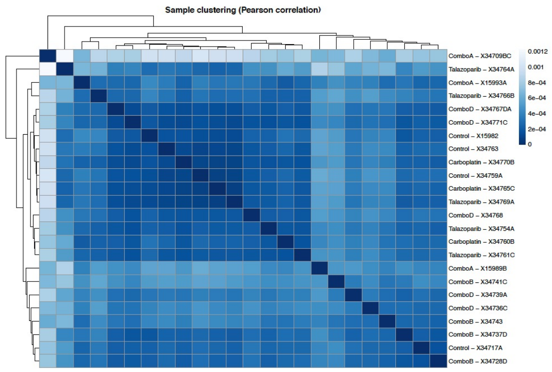


Combo A: Seq. T->C; Combo B: Seq. C->T, Combo D: Conc. T+C

B

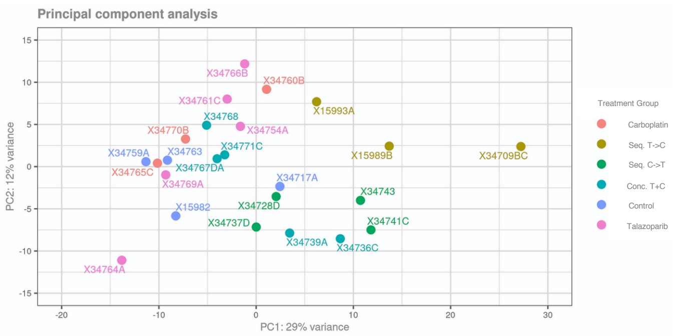


C

| Treatment Group Vs. Control | Carboplatin | Talazoparib | Conc. T+C | Seq. C->T | Seq.T->C |
|---|---|---|---|---|---|
| No. of significant gene sets upregulated* | 3 | 0 | 37 | 0 | 1 |
| No. of significant gene sets downregulated* | 0 | 1 | 1 | 0 | 9 |

| Seq. T->C Vs. Treatment Group | Carboplatin | Talazoparib | Conc. T+C | Seq. C->T |
|---|---|---|---|---|
| No. of significant gene sets upregulated* | 0 | 0 | 0 | 0 |
| No. of significant gene sets downregulated* | 14 | 16 | 13 | 11 |

D

Comparison of Conc. T+C versus Control

| Pathway | Enrichment Score (ES) | Normalized Enrichment Score (NES) | Nominal P-value | FDR q-value |
|---|---|---|---|---|
| KEGG_OTHER_GLYCAN_DEGRADATION | -0.704 | -1.683 | 0.021 | 0.210 |

E

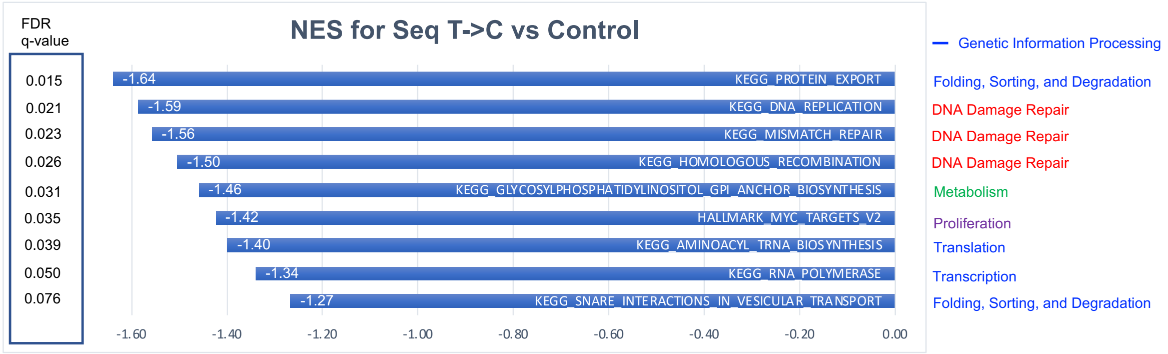


**Supplementary Fig. 11. Most differentially enriched pathways with Seq. T->C in comparison to control.** RNAseq analysis was performed from 5 representative FFPE samples of metastatic lung tissue from MDAMB231 orthotopic xenografts. Demonstrated above is the human cancer tissue component. After readcount normalization with DESeq2 v1.30, sample clustering and principal component analysis was performed (A,B). Preranked gene set enrichment analysis (GSEA) (GenePattern v.3.9.11) was performed to identify gene sets significantly up or downregulated while comparing treatment groups (C,D). For pathways that were statistically significant *(FDR<0.25), horizontal bar graphs indicating normalized enrichment scores and FDR q-values for each pathway (E).

**Supplementary Figure 12**

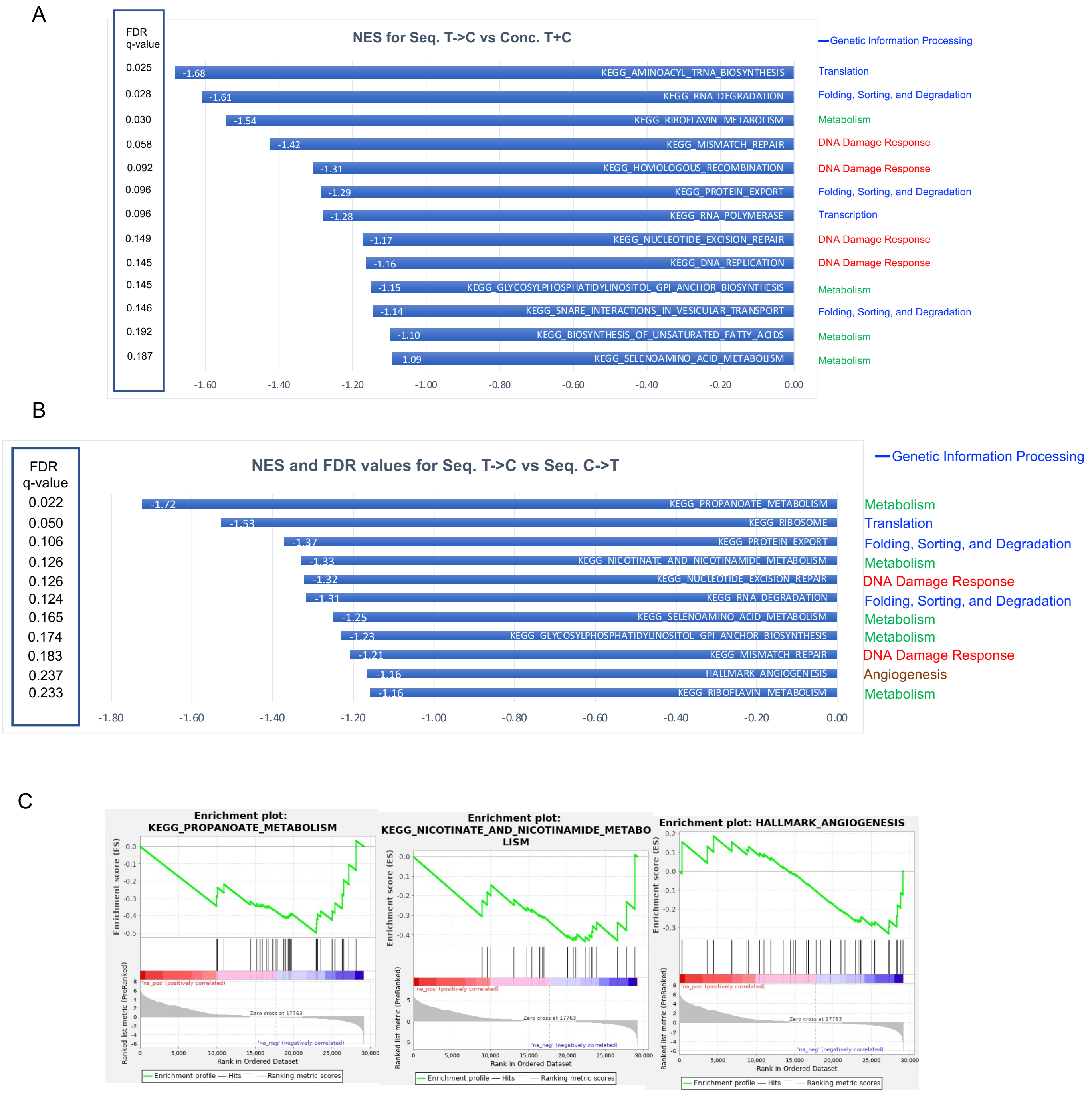


**Supplementary Fig. 12. Differentially enriched pathways with Seq. T->C in comparison to A) Conc. T+C, and (B,C) Seq. C->T.** RNAseq analysis was performed from 5 representative FFPE samples of metastatic lung tissue from MDAMB231 orthotopic xenografts. Demonstrated above is the human cancer tissue component. Preranked gene set enrichment analysis (GSEA) (GenePattern v.3.9.11) was performed to identify gene sets downregulated while comparing combination treatment groups with Seq. T->C for pathways with. For pathways that were statistically significant *(FDR<0.25), horizontal bar graphs indicating normalized enrichment scores and FDR q-values for each pathway (A,B). Representative enrichment plots for the downregulated pathways for Seq. T->C in comparison to Seq. C->T are demonstrated in (C).
