# Supplementary material for: Sequential targeting of PARP with carboplatin inhibits primary tumour growth and distant metastasis in triple-negative breast cancer
Source: Br J Cancer. 2023 Mar 20;128(10):1964–75. doi: 10.1038/s41416-023-02226-w (PMC10147920; doi:10.1038/s41416-023-02226-w)
Supplement: Supplementary file 3 — Supplementary Table 1 [file 41416_2023_2226_MOESM3_ESM.pdf]

**Supplementary Table 1.** Molecular features of breast cancer cell line panel with gene cluster status, breast cancer subtypes, and mutation status.

| | **MDAMB436** | **HCC1395** | **HCC1806** | **MX1**[1,2] | **SUM149PT**[3] | **BT20** | **HCC38** | **HCC1937**[4] | **HCC1187** | **HCC1143** | **MDAMB231** | **HCC2185** | **BT549** | **HS578T** |
|---|---|---|---|---|---|---|---|---|---|---|---|---|---|---|
| **Gene Cluster**[5] | BaB | B | BaA | NA | BaB | BaA | BaB | BaA | BaA | BaA | BaB | Lu | BaB | BaB |
| **Subtype**[4] | MSL | U | BL2 | NA | BL2 | U | BL1 | BL1 | IM | BL1 | MSL | LAR | M | MSL |
| **Subtype**[6] | CL | CL | B | NA | B | B | CL | B | B | B | CL | Lu | CL | CL |
| **Tp53**[MUT] | ■ | ■ | ■ | | ■ | ■ | ■ | ■ | ■ | ■ | ■ | | ■ | ■ |
| **BRCA1**[MUT] | ■ | | | ■ | ■ | | | ■ | | | | | | |
| **BRCA2**[MUT] | | ■ | | ■ | | | | | | | | | | |
| **PTEN-def**[7,8] | ■ | ■ | | | ■ | ■ | ■ | ■ | | | | | ■ | |
| **ATM**[MUT] | | | | | | ■ | | | | | ■ | | | |
| **ATR**[MUT] | | | | | | | | | | | | | ■ | |

Abbreviations: BaB, basal B; B, basal; BaA, basal A; NA, not available; Lu, luminal, MSL, mesenchymal stem-like; U, unclassified; BL1, basal-like 1; BL2, basal-like 2; IM, LAR, luminal androgen receptor; M, mesenchymal; CL, claudin-low


**References**

1. Donawho CK, Luo Y, Luo Y, et al. ABT-888, an orally active poly(ADP-ribose) polymerase inhibitor that potentiates DNA-damaging agents in preclinical tumor models. *Clinical Cancer Res*. 2007;13:2728-37.
2. Nair RS, Kumar JM, Jose J, et al. Increased sensitivity of BRCA defective triple negative breast tumors to plumbagin through induction of DNA Double Strand Breaks (DSB). *Scientific Reports* 2016;6:26631.
3. Wasielewski, M., Elstrodt, F., Klijn, J.G. *et al.* Thirteen new *p53* gene mutants identified among 41 human breast cancer cell lines. *Breast Cancer Res Treat* **99,** 97–101 (2006). https://doi.org/10.1007/s10549-006-9186-z.
4.Lehmann BD, Bauer JA, Chen X, et al. Identification of human triple-negative breast cancer subtypes and preclinical models for selection of targeted therapies. *The Journal of Clinical Investigation* 2011;121:2750-67.
5. Neve RM, Chin K, Fridlyand J, et al. A collection of breast cancer cell lines for the study of functionally distinct cancer subtypes. *Cancer Cell* 2006;10:515-27.
6. Heiser LM, Sadanandam A, Kuo WL, et al. Subtype and pathway specific responses to anticancer compounds in breast cancer. *Proceedings of the National Academy of Sciences of the United States of America* 2012;109:2724-9.
7. Tang YC, Ho SC, Tan E, et al. Functional genomics identifies specific vulnerabilities in PTEN-deficient breast cancer. *Breast Cancer Res*. 2018;20(1):22. doi:10.1186/s13058-018-0949-3
8. Brunner A, Suryo Rahmanto A, Johansson H, et al. PTEN and DNA-PK determine sensitivity and recovery in response to WEE1 inhibition in human breast cancer. *Elife*. 2020;9:e57894. Published 2020 Jul 6. doi:10.7554/eLife.57894
9. Tate JG, Bamford S, Jubb HC, et al. COSMIC: the Catalogue Of Somatic Mutations In Cancer, *Nucleic Acids Research*, Volume 47, Issue D1, 08 January 2019, Pages D941–D947, https://doi.org/10.1093/nar/gky1015
